# High-density lipoprotein cholesterol concentration and acute kidney injury after noncardiac surgery

**DOI:** 10.1186/s12882-020-01808-7

**Published:** 2020-04-25

**Authors:** Yan Zhou, Hong-Yun Yang, Hui-Li Zhang, Xiao-Jin Zhu

**Affiliations:** 1 Department of Anesthesiology and Critical Care Medicine, Peking University First Hospital, Beijing, 100034 China; 2 Department of Laboratory, Peking University First Hospital, Beijing, 100034 China; 3 Department of information center, Peking University First Hospital, Beijing, 100034 China

**Keywords:** Acute kidney injury, High-density lipoprotein cholesterol, Noncardiac surgery, Risk factors

## Abstract

**Background:**

Abnormal High-density Lipoprotein Cholesterol Concentration is closely related to postoperative acute kidney injury (AKI) after cardiac surgeries. The purpose of this study was to analyze the relationship between High-density Lipoprotein Cholesterol Concentration and acute kidney injury after non-cardiac surgeries.

**Method:**

This was a single-center cohort study for elective non-cardiac non-kidney surgery from January 1, 2012, to December 31, 2017. The endpoint was the occurrence of acute kidney injury (AKI) 7 days postoperatively in the hospital. Preoperative serum High-density Lipoprotein Cholesterol Concentration was examined by multivariate logistic regression models before and after propensity score weighting analysis.

**Results:**

Of the 74,284 surgeries, 4.4% (3159 cases) suffered acute kidney injury. The odds ratio for HDL (0.96–1.14 as reference, < 0.96, 1.14–1.35, > 1.35) was 1.28 (1.14–1.41), *P* < 0.001; 0.91 (0.80–1.03), *P* = 0.150; 0.75 (0.64–0.85), P < 0.001, respectively. Using a dichotomized cutoff point for propensity analysis, Preoperative serum HDL <  1.03 mmol/L (> 1.03 as reference) was associated with increased risk of postoperative AKI, with odds ratio 1.40 (1.27 ~ 1.52), *P* < 0.001 before propensity score weighting, and 1.32 (1.21–1.46), P < 0.001 after propensity score weighting. Sensitivity analysis with other cut values of HDL showed similar results.

**Conclusions:**

Using multivariate regression analyses before and after propensity score weighting, in addition to multiple sensitivity analysis methods, this study found that following non-cardiac surgery, low HDL cholesterol levels were independent risk factors for AKI.

## Key points

**Question**: Is High-density Lipoprotein Cholesterol Concentration associated with acute kidney injury after non-cardiac surgeries?

**Finding**: The odds ratio for HDL (0.96–1.14 as reference, < 0.96, 1.14–1.35, > 1.35) was 1.28 (1.14–1.41), *P* < 0.001; 0.91 (0.80–1.03), *P* = 0.150; 0.75 (0.64–0.85), P < 0.001, respectively.

**Meaning**: low HDL cholesterol levels were independent risk factors for AKI after non-cardiac surgery.

## Background

Acute kidney injury (AKI) is a common complication after both cardiac and non-cardiac surgery. The prevalence of AKI varies greatly, from 1.1% of patients after the minimally invasive procedures to 17.9% of patients following major surgeries [1–9]. The patients’ mortality rate increases significantly with the occurrence of AKI.^3–5^ There are many risk factors associated with AKI. These include factors that cannot be modified, for example, gender, body mass index (BMI), age, time and complexity of the surgery. And factors that can be modified, for example, preoperative albumin levels, duration of hypotension, colloid and dexmedetomidine use [1–7].

Recent studies found that after major cardiac surgery the concentration of High-density Lipoprotein Cholesterol Concentration (HDL) was associated with AKI[10]. However, no studies were found regarding AKI after non-cardiac surgery. The present research aims to determine the relationship between AKI and the concentration of and AKI after non-cardiac surgery.

## Methods

### Study design

This study was approved by the Peking University First Hospital Ethics Committee, and the requirement for written informed consent was waived.

### Data source and study patients

This study used data obtained from the perioperative database of Peking University First Hospital, which contains the perioperative information of inpatients from 2012 onward. This study analyzed data from adults (age ≥ 18 years old) who underwent elective non-cardiac surgery between January 1, 2012, and December 31, 2017. Non-cardiac non-kidney surgery was identified based on the International Classification of Diseases and Procedures, Ninth Clinical Revision Revision volume 3 (ICD-9-v3). All surgeries other than those with their ICD codes listed were defined as “other.” (Table S1 in the Supplementary Appendix).

Patients were excluded from the study based on the following criteria, those that underwent cardiac surgeries, kidney surgeries, obstetric surgeries, local infiltration anesthesia, and missing perioperative data. Also, patients with more than one operation within a year (including reopening of surgical cases) were excluded.

The patients’ preoperative serum cholesterol levels were obtained from the laboratory database and linked to the hospital’s perioperative database. Results were categorized according to the day on which patients’ were operated on. The mean value (3 month time frame) was calculated for each patient with more than one preoperative result.

### Study ENDPOINTS

The endpoint was any patient with AKI within 7 days in the hospital. This study used KDIGO as the criteria for AKI, which was defined by the patient’s postoperative serum creatinine increase to not less than 26.5 μmol/l within 48 h, or 1.5 times from the baseline within 7 days after surgery, or initialization of blood dialysis. As the serum creatinine level fluctuates much postoperatively and could cause an inaccurate estimate of Glomerular Filtration Rate (eGFR), this study did not define AKI based on the GFR value or urine output.

### Statistical analysis

According to previous studies, the incidence of postoperative AKI would be 1.1–17.9% in patients that elected to have surgery [1–9]. We expected the patients with an abnormal serum cholesterol level to have an OR of 1.20 when compared with the normal serum cholesterol level. With significance set at 0.05 and the power set at 90%, the calculated sample size needed to compare two proportions was 3206 patients in each group.

For the comparative analysis, patients were divided into two groups according to the occurrence of postoperative AKI. Continuous variables with a normal distribution were compared using the Student t-test, and those with non-normal distribution were compared with the Mann-Whitney U-test. The Kolmogorov-Smirnov test was used to determine whether the data were normally distributed or not. Categorical variables were compared using the Chi-Square test or continuity corrected Chi-Square test. Rank variables were compared using the Kruskal–Wallis H-test.

### Logistic regression was used to detect any association between the concentrations of HDL and AKI

A logistic regression model was constructed using the following formula:

$$ \mathrm{AKI}=\mathrm{HDL}+\mathrm{confounders} $$

Confounders (covariates) were the same in both the logistic regression and generalized additive models, except for the HDL levels. Confounders were assessed based on a priori knowledge and other studies [1–9, 11]. The following covariates were considered: sex, age, BMI, revised cardiac risk index grade, surgery duration, anesthesia type, cancer surgery, intraoperative blood transfusion, surgical complexity (Modified John Hopkins hospital criteria, MJHSC, [12]. Table-S5 in Appendix supplement 1) and preoperative serum albumin and serum creatinine, anesthesiologist’s experience, intraoperative dexmedetomidine and colloid use.

### The propensity score weighting analysis

Due to the huge systematic differences, this study balanced the patients with preoperative HDL below or above 1.03 mmol/L (the widely accepted threshold for cardiovascular risk [13]) by propensity score weighting. Propensity score weighting is a method to diminish the effect of measured confounding factors and to get a less biased result in observational studies. In the present study, propensity score weights were calculated by using gradient boosted regression models,[14, 15] in which high or low preoperative HDL was the dependent variable, and vectors of the following (age, gender, body mass index, revised cardiac risk index, surgery duration, anesthesia type, cancer surgery, intraoperative blood transfusion, surgical complexity, type of surgery classified by site, anesthesiologist’s experience, severe intraoperative hypotension, preoperative coronary heart disease, arrhythmia, cerebral infarction, diabetes, chronic kidney disease, preoperative creatinine, cholesterol components, intraoperative dexmedetomidine and colloid use) were the independent variables. Compared to the inverse probability of exposure weighting method (IPEW), [16] the propensity score weights calculated by the gradient boosted regression models do not need to consider co-linearity and often get better balancing performance [14, 15]. Using the weights for the originally observed cohorts could create two new cohorts with the number of patients differing from the original, and may not be an integer (each patient was multiplied by a specific weight defined by the gradient boosted regression models). The logistic analysis with the propensity score weighting could lead to less biased results, i.e., a quasi-randomized study.

### Statistical packages

All data management and statistical analysis were performed using the R programming language (v.3.5.2).

## Result

### Study population

This study identified 60,772 non-cardiac non-kidney and non-obstetric elective surgeries from 57,983 unique patients between January 1, 2012, and December 31, 2017 (Figure-S1 in the supplement). A total of 2649 (4.4%) cases had postoperative AKI, of which, 2192,152, and 305 cases were AKI grades 1, 2 and 3, respectively. Compared to those without it, patients with postoperative AKI were older, were more likely to be male, have a low body mass index, have a co-existing disease, have high blood pressure, and to be in a lengthy surgery (Tables 1 and 2). (Table S1 in the Supplementary)

**Table 1:** Demographic characteristics and preoperative comorbidities

characteristic	ALL (***n*** = 60,772)	No AKI (***n*** = 58,123)	AKI (***n*** = 2649)	***P*** value
Age	56.2 ± 15.8	55.9 ± 15.8	62.5 ± 14.5	< 0.001
Gender, female	37,226(49.2%)	36,120(49.8%)	1106(35.0%)	< 0.001
Body mass index, kg/m^2^	24.5 ± 3.7	24.5 ± 3.7	24.5 ± 4.0	0.936
Co-existing disease				
hypertension	21,236(28.0%)	19,889(27.4%)	1347(42.6%)	< 0.001
Coronary artery disease	3938(5.2%)	3588(4.9%)	350(11.1%)	< 0.001
Heart failure	704(0.9%)	575(0.8%)	129(4.1%)	< 0.001
Stroke	3092(4.1%)	2833(3.9%)	259(8.2%)	< 0.001
diabetes mellitus	9184(12.1%)	8516(11.7%)	668(21.1%)	< 0.001
Renal insufficiency	1009(1.3%)	561(0.8%)	448(14.2%)	< 0.001
Regular statin therapy^a^	900(0.8%)	822(0.8%)	78(1.6%)	< 0.001
ASA				
I	18,472(24.4%)	18,181(25.1%)	291(9.2%)	< 0.001
II	51,065(67.6%)	49,093(67.8%)	1972(62.6%)	
III	5900(7.8%)	5057(7.0%)	843(26.8%)	
IV	138(0.2%)	95(0.1%)	43(1.4%)	

Results reported as mean ± SD or n (%)

a: Only regular statin therapy with more than 3 months was count

**Table 2:** perioperative parameters

characteristic	ALL (n = 60,772)	No AKI (n = 58,123)	AKI (n = 2649)	*P* value
Anesthesia duration, min	189.7 ± 123.0	187.2 ± 121.3	244.7 ± 146.0	< 0.001
Anesthesia type				< 0.001
General anesthesia	48,273(77.9%)	46,117(77.8%)	2156(81.0%)	
General anesthesia + epidural/nerve block	4044(6.5%)	3791(6.4%)	253(9.5%)	
Neuraxial or nerve block	9642(15.6%)	9388(15.8%)	254(9.5%)	
Infusion volume, ml	1100(750–1800)	1100(700–1700)	1600(1100–2600)	< 0.001
Crystal, ml	1100(600–1600)	1000(600–1600)	1300(1000–2100)	< 0.001
Colloid, ml	0(0–500)	0(0–500)	0(0–500)	< 0.001
Estimated blood loss, ml	0(0–50)	0(0–50)	30(0–200)	< 0.001
Intraoperative blood infusion	5725(9.2%)	5263(8.9%)	462(17.3%)	< 0.001
Urine, ml	0(0–300)	0(0–300)	100(0–400)	< 0.001
Surgery time, min	117.5 ± 123.9	115.8 ± 121.6	156.0 ± 163.2	< 0.001
Surgery type				< 0.001
Eye/ear/throat	3089(5.0%)	3054(5.2%)	35(1.3%)	
Integumentary	2221(3.6%)	2181(3.7%)	40(1.5%)	
Genital/urinary	16,264(26.2%)	15,150(25.5%)	1114(41.8%)	
Musculoskeletal	7390(11.9%)	7153(12.1%)	237(8.9%)	
Nervous	3545(5.7%)	3412(5.8%)	133(5.0%)	
Vascular	2233(3.6%)	2160(3.6%)	73(2.7%)	
Digestive	20,936(33.8%)	20,094(33.9%)	842(31.6%)	
Respiratory	3528(5.7%)	3395(5.7%)	133(5.0%)	
Other	2753(4.4%)	2697(4.5%)	56(2.1%)	
Intraoperative mean HR, bpm	65.7 ± 9.8	65.6 ± 9.7	67.4 ± 10.9	< 0.001
Baseline SBP, mmHg	127.8 ± 17.7	127.6 ± 17.6	132.1 ± 18.7	< 0.001
Baseline DBP, mmHg	76.5 ± 10.9	76.5 ± 10.8	77.4 ± 11.9	< 0.001
KDIGO AKI grade				
III	306(0.8%)	–	306(11.5%)	< 0.001
II	154(0.4%)	–	154(5.8%)	< 0.001
I	2203(5.8%)	–	2203(82.7%)	< 0.001

### Results of the logistic regression to detect any association between the concentrations of HDL and AKI

The results of the multivariate logistic regression showed an association between low HDL and postoperative AKI. The odds ratio for HDL (0.96–1.14 as reference, < 0.96, 1.14–1.35, > 1.35) was 1.28 (1.14–1.41), *P* < 0.001; 0.91 (0.80–1.03), *P* = 0.150; 0.75 (0.64–0.85), P < 0.001, respectively. (Table 3)

**Table 3:** Association detection with logistic regression models and causal inference after propensity score weighting analysis

	Cut value (mmol/l)	Odds ratio by multivariate logistic regression	P value	Before or after propensity score weighting
Model 1^a^	0.96–1.14	reference		before
	< 0.96	1.28 (1.14 ~ 1.41)	<.001	
	1.14–1.35	0.91 (0.80 ~ 1.03)	0.15	
	> 1.35	0.75 (0.64 ~ 0.85)	<.001	
Model 2^b^	< 1.03 vs ≥ 1.03	1.40 (1.27 ~ 1.52)	<.001	before
Model 3^c^	< 1.03 vs ≥ 1.03	1.32 (1.21–1.46)	<.001	after

a: multivariate logistic regression model without propensity score weighting, the HDL was cut by quantile

b: multivariate logistic regression model without propensity score weighting, the HDL was cut by 1.03 mmol/l.

c: multivariate logistic regression model after propensity score weighting, the HDL was cut by 1.03 mmol/l.

### Results of the propensity score weighting analysis

After the propensity score weighting, the data set was divided into two groups i.e. patients with an HDL level above and below the predetermined HDL cholesterol value of 1.03 mmol/L which was obtained from previously published studies [13]. Also, the logistic odds ratio was calculated. The odds ratio for HDL (> 1.03 as reference) was 1.32 (1.21 - 1.46), *P* < 0.001. (Table 3) (Tables S2, S3, S4 in the Supplementary).

### Sensitivity analysis

Different cutoff points were used to reanalyze the association between the concentration of HDL and AKI, and similar results to the propensity score weighting analysis were found. (Table S5 in the Supplementary).

## Discussion

This study showed that postoperative AKI was 4.4% of adult patients undergoing elective non-cardiac surgery. Multi-variable adjustment before or after propensity score weighting showed that low concentrations of HDL was strongly associated with AKI following non-cardiac surgery. After adjusting for other risk factors, low HDL was still an independent risk factor for cardiovascular disease [17–22]. However, an increase in HDL did not reduce cardiovascular events, and there was no causal relationship between them [23–26]. The MESA cohort study found that HDL particles were more predictive of cardiovascular events than HDL cholesterol, suggesting that certain structures and features of HDL play key roles [21]. There appeared to be no causal relationship between HDL cholesterol levels and cardiovascular disease.

This study found that after non-cardiac surgery, preoperative HDL levels were independent risk factors for postoperative AKI. After the balancing of possible confounding factors and other cholesterol and triglycerides levels, the results still showed that HDL was closely related to postoperative AKI.

The mechanism by which HDL protects the patient against postoperative AKI is not clear. The mechanisms related to HDL and cardiovascular events include macrophage cholesterol efflux [24, 27]; promoting maintenance of endothelial function [28, 29]; protection against oxidation of LDL [30, 31]; protection against inflammation [32]; immunomodulation [33]; and finally, via a variety of actions, interfering with the thrombotic component of atherosclerosis [34–37]. These mechanisms may also play a role in the prevention of AKI. There may be other mechanisms specific to the kidney currently not known and needing further research. Studies have reported that the dysfunction of HDL’s anti-inflammation, anti-oxidation, and endothelial protection are associated with an increased risk of chronic renal disease [38–40].

Researchers have found that serum HDL is high, antioxidant enzymes and oxidative stress levels are reduced, which was related to receptors acting as pro-oxidant lipids [41–44]. Moreover, high HDL was related to decreased expression of adhesion molecules, increased nitric oxide, and decreased damage to the endothelium [42, 45, 46]. In patients with chronic kidney disease, the function of their HDL as an anti-inflammatory, antioxidant and protector of their endothelial, was found to be decrease [40, 47–49]. These processes (systemic inflammation, oxidative stress, and endothelial dysfunction and damage) all play a key role in postoperative renal dysfunction and AKI.

Our study found that the preoperative use of a statin did not reduce postoperative AKI. This is consistent with previous multiple high-quality studies [50–52]. Recently, one study suggested that long-term use of a statin can enhance the protection by HDL [10]. These contradictory results suggest that the protective effect of statins on the kidney is not direct and requires further research.

AKI results in a higher mortality rate, longer hospitalization, and increased costs. It is of paramount importance to focus on the possible risk factors related to AKI that can be managed. For those at high risk, earlier lab tests, medication selection, and measures to protect the kidney may improve the prognosis with minimal cost and medical resources required.

Some research found high HDL was related to longer life expectancy. The importance and mechanism of HDL throughout the human body still not fully understood. Our research proposes a possible research direction on HDL.

Although the authors tried their best to implement the best research methods and improve the quality of the database, various shortcomings and errors were still inevitable, including: 1. This study was a retrospective cohort study, which may result in inaccuracies. 2. Postoperative laboratory tests and exams were not performed on every patient but were based on clinical observations when symptoms and signs were suspected, which may result in an underestimation of the primary outcome rate. A few patients were discharged from the hospital within 7 days of their surgeries, without any subsequent following. There may be a small number of patients with primary outcome events that were not detected.

## Conclusion

Using multivariate regression analyses before and after propensity score weighting in addition to multiple sensitivity analysis methods, this study found that following non-cardiac surgery, low HDL cholesterol levels were independent risk factors for AKI.

## Supplementary information

**Additional file 1.**

## Data Availability

The data that support the findings of this study are available from [Peking University First Hospital] but restrictions apply to the availability of these data, which were used under license for the current study, and so are not publicly available. Data are however available from the authors upon reasonable request and with permission of [Peking University First Hospital].

## Abbreviations

AKI: Acute kidney injury
LDL: Low-density lipoprotein cholesterol
HDL: High-density lipoprotein cholesterol
TG: Triglycerides
ICD-9-v3: INTERNATIONAL Classification of diseases and procedures, Ninth Revision Clinical Revision volume 3
ICD: International Classification of diseases and procedures
eGFR: Estimated Glomerular Filtration Rate
MJHSC: Modified John Hopkins hospital criteria

## References

[1] Sun LY, Wijeysundera DN, Tait GA, Beattie WS (2015). Association of intraoperative hypotension with acute kidney injury after elective noncardiac surgery. Anesthesiology.

[2] Ng RRG, Chew STH, Liu W, Shen L, Ti LK (2014). Identification of modifiable risk factors for acute kidney injury after coronary artery bypass graft surgery in an Asian population. J Thorac Cardiovasc Surg.

[3] Lee E-H, Kim W-J, Kim J-Y, Chin J-H, Choi D-K, Sim J-Y, Choo S-J, Chung C-H, Lee J-W, Choi I-C (2016). Effect of exogenous albumin on the incidence of postoperative acute kidney injury in patients undergoing off-pump coronary artery bypass surgery with a preoperative albumin level of less than 4.0 g/dl. J Anesthesiology.

[4] Lee E-H, Baek S-H, Chin J-H, Choi D-K, Son H-J, Kim W-J, Hahm K-D, Sim J-Y, Choi I-C (2012). Preoperative hypoalbuminemia is a major risk factor for acute kidney injury following off-pump coronary artery bypass surgery. J Intens Care Med.

[5] Kim M, Brady JE, Li G (2014). Variations in the risk of acute kidney injury across intraabdominal surgery procedures. J Anesthesia Analgesia.

[6] Goren O, Levy A, Cattan A, Lahat G, Matot I (2017). Acute kidney injury in pancreatic surgery; association with urine output and intraoperative fluid administration. Am J Surg.

[7] Garg AX, Kurz A, Sessler DI, Cuerden M, Robinson A, Mrkobrada M, Parikh CR, Mizera R, Jones PM, Tiboni M (2014). Perioperative aspirin and clonidine and risk of acute kidney injury: a randomized clinical trial. J Jama.

[8] Futier E, Lefrant J-Y, Guinot P-G, Godet T, Lorne E, Cuvillon P, Bertran S, Leone M, Pastene B, Piriou V (2017). Effect of individualized vs standard blood pressure management strategies on postoperative organ dysfunction among high-risk patients undergoing major surgery: a randomized clinical trial. J Jama.

[9] Bress AP, Kramer H, Khatib R, Beddhu S, Cheung AK, Hess R, Bansal VK, Cao G, Yee J, Moran AE (2017). Potential deaths averted and serious adverse events incurred from adoption of the SPRINT (systolic blood pressure intervention trial) intensive blood pressure regimen in the United States: projections from NHANES (National Health and nutrition examination survey). J Circulation.

[10] Smith LE, Smith DK, Blume JD, Linton MRF, Billings IV, Frederic T (2017). High-Density Lipoprotein Cholesterol Concentration and Acute Kidney Injury After Cardiac Surgery. J Am Heart Assoc.

[11] Wilson T, Quan S, Cheema K, Zarnke K, Quinn R, de Koning L, Dixon E, Pannu N, James MT (2015). Risk prediction models for acute kidney injury following major noncardiac surgery: systematic review. J Nephrology Dial Transplant.

[12] Donati A, Ruzzi M, Adrario E, Pelaia P, Coluzzi F, Gabbanelli V, Pietropaoli P (2004). A new and feasible model for predicting operative risk. Br J Anaesth.

[13] Superko HR, Pendyala L, Williams PT, Momary KM, King SB, Garrett B (2012). High-density lipoprotein subclasses and their relationship to cardiovascular disease. J Clin Lipidol.

[14] Ridgeway G, McCaffrey D, Morral A, Burgette L, Griffin BA, CA: RAND Corporation (2006). Toolkit for Weighting and Analysis of Nonequivalent Groups: A tutorial for the twang package.

[15] McCaffrey DF, Griffin BA, Almirall D, Slaughter ME, Ramchand R, Burgette LF (2013). A tutorial on propensity score estimation for multiple treatments using generalized boosted models. Stat Med.

[16] Jones PM, Cherry RA, Allen BN, Bray Jenkyn KM, Shariff SZ, Flier S, Vogt KN, Wijeysundera DN (2018). Association between handover of anesthesia care and adverse postoperative outcomes among patients undergoing major surgery. JAMA.

[17] Sacks FM, Tonkin AM, Craven T, Pfeffer MA, Shepherd J, Keech A, Furberg CD, Braunwald E (2002). Coronary heart disease in patients with low LDL-cholesterol: benefit of pravastatin in diabetics and enhanced role for HDL-cholesterol and triglycerides as risk factors. J Circulation.

[18] Barter P, Gotto AM, LaRosa JC, Maroni J, Szarek M, Grundy SM, Kastelein JJP, Bittner V, Fruchart J-C (2007). HDL cholesterol, very low levels of LDL cholesterol, and cardiovascular events. N Eng J Medicine.

[19] Acharjee S, Boden WE, Hartigan PM, Teo KK, Maron DJ, Sedlis SP, Kostuk W, Spertus JA, Dada M, Chaitman BR (2013). Low levels of high-density lipoprotein cholesterol and increased risk of cardiovascular events in stable ischemic heart disease patients: a post-hoc analysis from the COURAGE trial (clinical outcomes utilizing revascularization and aggressive drug evaluation). J Am Coll Cardiol.

[20] Mora S, Glynn RJ, Ridker PM (2013). High-density lipoprotein cholesterol, size, particle number, and residual vascular risk after potent statin therapy. Circulation.

[21] Mackey RH, Greenland P, Goff DC, Lloyd-Jones D, Sibley CT, Mora S (2012). High-density lipoprotein cholesterol and particle concentrations, carotid atherosclerosis, and coronary events: MESA (multi-ethnic study of atherosclerosis). J Am Coll Cardiol.

[22] Asztalos BF, Collins D, Horvath KV, Bloomfield HE, Robins SJ, Schaefer EJ (2008). Relation of gemfibrozil treatment and high-density lipoprotein subpopulation profile with cardiovascular events in the Veterans Affairs High-Density Lipoprotein Intervention Trial. Metabolism.

[23] Briel M, Ferreira-Gonzalez I, You JJ, Karanicolas PJ, Akl EA, Wu P, Blechacz B, Bassler D, Wei X, Sharman A, Whitt I (2009). Association between change in high density lipoprotein cholesterol and cardiovascular disease morbidity and mortality: systematic review and meta-regression analysis. BMJ.

[24] Rosenson RS, Brewer J, Bryan H, Davidson WS, Fayad ZA, Fuster V, Goldstein J, Hellerstein M, Jiang X-C, Phillips MC, Rader DJ (2012). Cholesterol efflux and atheroprotection: advancing the concept of reverse cholesterol transport. Circulation.

[25] Haase CL, Tybjærg-Hansen A, Grande P, Frikke-Schmidt R (2010). Genetically elevated apolipoprotein AI, high-density lipoprotein cholesterol levels, and risk of ischemic heart disease. J Clin Endocrinol Metab.

[26] Haase CLT-HA, Ali Qayyum A, Schou J, Nordestgaard BG, Frikke-Schmidt R (2012). LCAT, HDL cholesterol and ischemic cardiovascular disease: a Mendelian randomization study of HDL cholesterol in 54,500 individuals. J Clin Endocrinol Metab.

[27] Rosenson RSBJH, Ansell B, Barter P, Chapman MJ, Heinecke JW, Kontush A, Tall AR, Webb NR (2013). Translation of high-density lipoprotein function into clinical practice: current prospects and future challenges. Circulation.

[28] Kuhn FEME, Satler LF, Reagan K, Lu DY, Rackley CE (1991). Effects of high-density lipoprotein on acetylcholine-induced coronary vasoreactivity. Am J Cardiol.

[29] Oram JF, Johnson CJ, Brown TA (1987). Interaction of high density lipoprotein with its receptor on cultured fibroblasts and macrophages. Evidence for reversible binding at the cell surface without internalization. J Biol Chem.

[30] Kontush A, Chantepie S, Chapman MJ (2003). Small, dense HDL particles exert potent protection of atherogenic LDL against oxidative stress. Arterioscler Thromb Vasc Biol.

[31] Soran HSJ, Liu Y, Durrington PN (2015). How HDL protects LDL against atherogenic modification: paraoxonase 1 and other dramatis personae. Curr Opin Lipidol.

[32] Barter PJNS, Rye KA, Anantharamaiah GM, Navab M, Fogelman AM (2004). Antiinflammatory properties of HDL. Circ Res.

[33] Murphy AJ, Akhtari M, Tolani S, Pagler T, Bijl N, Kuo CL, Wang M, Sanson M, Abramowicz S, Welch C, Bochem AE (2011). ApoE regulates hematopoietic stem cell proliferation, monocytosis, and monocyte accumulation in atherosclerotic lesions in mice. J Clin Invest.

[34] Saku K, Ahmad M, Glas-Greenwalt P, Kashyap ML (1985). Activation of fibrinolysis by apolipoproteins of high density lipoproteins in man. Thromb Res.

[35] Griffin JH, Kojima K, Banka CL, Curtiss LK, Fernández JA (1999). High-density lipoprotein enhancement of anticoagulant activities of plasma protein S and activated protein C. J Clin Invest.

[36] Epand RM, Stafford A, Leon B, Lock PE, Tytler EM, Segrest JP, Anantharamaiah GM (1994). HDL and apolipoprotein AI protect erythrocytes against the generation of procoagulant activity. Arterioscler Thromb.

[37] Aoyama T, Yui Y, Morishita H, Kawai C (1990). Prostaglandin I2 half-life regulated by high density lipoprotein is decreased in acute myocardial infarction and unstable angina pectoris. Circulation.

[38] Yamamoto S, Yancey PG, Ikizler TA, Jerome WG, Kaseda R, Cox B, Bian A, Shintani A, Fogo AB, Linton MRF (2012). Dysfunctional high-density lipoprotein in patients on chronic hemodialysis. J Am Coll Cardiol.

[39] Shroff R, Speer T, Colin S, Charakida M, Zewinger S, Staels B, Chinetti-Gbaguidi G, Hettrich I, Rohrer L, O’Neill F (2014). HDL in children with CKD promotes endothelial dysfunction and an abnormal vascular phenotype. J Am Soc Nephrol.

[40] Kalantar-Zadeh K, Kopple J, Kamranpour N, Fogelman A, Navab M (2007). HDL-inflammatory index correlates with poor outcome in hemodialysis patients. Kidney Int.

[41] Murphy AJ, Woollard KJ, Hoang A (2008). High-density lipoprotein reduces the human monocyte inflammatory response. Arterioscler Thromb Vasc Bio.

[42] Wan Ahmad W, Sakri F, Mokhsin A (2015). Low serum high density lipoprotein cholesterol concentration is an independent predictor for enhanced inflammation and endothelial activation. PLoS One.

[43] Navab M, Hama SY, Anantharamaiah G (2000). Normal high density lipoprotein inhibits three steps in the formation of mildly oxidized low density lipoprotein: steps 2 and 3. J Lipid Res.

[44] Navab M, Berliner JA, Subbanagounder G (2001). HDL and the inflammatory response induced by LDL-derived oxidized phospholipids. Arterioscler Thromb Vasc Biol.

[45] Yuhanna IS, Zhu Y, Cox BE (2001). High-density lipoprotein binding to scavenger receptor-BI activates endothelial nitric oxide synthase. Nat Med.

[46] Tso C, Martinic G, Fan W-H (2006). High-density lipoproteins enhance progenitor-mediated endothelium repair in mice. Arterioscler Thromb Vasc Biol.

[47] Shroff R, Speer T, Colin S (2014). HDL in children with CKD promotes endothelial dysfunction and an abnormal vascular phenotype. J Am Soc Nephrol.

[48] Jones PH, Nair R, Thakker KM (2012). Prevalence of dyslipidemia and lipid goal attainment in statin-treated subjects from 3 data sources: a retrospective analysis. J Am Heart Assoc.

[49] Yamamoto S, Yancey PG, Ikizler TA (2012). Dysfunctional high-density lipoprotein in patients on chronic hemodialysis. J Am Coll Cardiol.

[50] Zheng Z, Jayaram R, Jiang L, Emberson J, Zhao Y, Li Q, Du J, Guarguagli S, Hill M, Chen Z (2016). Perioperative rosuvastatin in cardiac surgery. N Engl J Med.

[51] Park JH, Shim J-K, Song J-W, Soh S, Kwak Y-L (2016). Effect of atorvastatin on the incidence of acute kidney injury following valvular heart surgery: a randomized, placebo-controlled trial. Intensive Care Med.

[52] Billings FT, Hendricks PA, Schildcrout JS, Shi Y, Petracek MR, Byrne JG, Brown NJ (2016). High-dose perioperative atorvastatin and acute kidney injury following cardiac surgery: a randomized clinical trial. JAMA.

