# Supplementary material for: High-density lipoprotein cholesterol concentration and acute kidney injury after noncardiac surgery
Source: BMC Nephrol. 2020 Apr 25;21:149. doi: 10.1186/s12882-020-01808-7 (PMC7183648; doi:10.1186/s12882-020-01808-7)
Supplement: Supplementary file 1 — Additional file 1. [file 12882_2020_1808_MOESM1_ESM.docx]

Supplementary Appendix

Yan Zhou, MD, PhD

**Table of Contents:**

Covariates

Definition of Pre-Existing Disease

Definition of Outcomes

Supplementary Tables

Supplementary Figure

**Covariates**

**Baseline and demographic data**

The following baseline demographic characteristics will be included in the analysis:

age, gender, body mass index.

**Definition of Pre-Existing Disease**

**Coronary artery disease:** Patients’ diagnosis with ICD code I21, I22, I23, I24, I25 were defined as Coronary artery disease.

**Myocardial infarction:** Patients’ diagnosis with ICD code I21, I22. Or max preoperative Troponin I more than 0.05 with clinical symptoms or new Electrocardiography abnormalities were defined as myocardial infarction.

**Hypertension:** Patients’ diagnosis with ICD code I10-I15, I11, E10.722, E10.723, E11.722, E11.723, E14.722, E14.723, E16.8x101, E16.8x102, N28.917, O10.001, O10.101, O10.201, O10.301, O10.401, O10.901, O10.902, O11xx01, O13xx01, O16xx01, O99.415, P29.201, T70.202 were defined as hypertension.

**Congestive heart failure:** Patients’ diagnosis with ICD code I50, I97.104, T81.810, I11.001, I13.201, I97.106, N18.820, O29.102, O74.202, O75.402, O89.102, O99.408, O99.423. Or max preoperative Brain natriuretic peptide more than 400pg/ml with signs and symptoms of heart failure were defined as congestive heart failure.

**Arrythmia:** Patients’ diagnosis with ICD code I44, I45, I46, I47, I48, I49 were defined as arrythmia.

**Stroke:** Patients’ diagnosis with ICD code I63, I64, I693-I698 were defined as Stroke

**Diabetes:** Patients’ diagnosis with ICD code E8, E9, E10, E11, E13 were defined as diabetes.

**Chronic renal disease:** Patients’ diagnosis with ICD code N18, N19 were defined as chronic renal disease.

**Anemia:** Patients’ diagnosis with ICD code D60, D61, D62, D63, D64 were defined as anemia.

**Revised cardiac risk index**

The revised cardiac risk index (rCRI) was defined by congestive heart failure (CHF), stroke, myocardial infarction, and diabetes using ICD-10 diagnostic codes; high-risk surgery was defined following the result of Schwarze’s work using ICD-9-v3 codes^1^; and laboratory data identifying maxium serum creatinine values greater than 133 μmol/l within 90 days before surgery. Insulin-dependent diabetes was extracted from database obtaining from preoperative evaluation. The revised cardiac risk index was categorized to: low risk (1 point), moderate risk (2 points), or high risk (≥3 points).

**Anti hypertensive medication:** Patients taking regular antihypertensive drugs were divided into alpha antagonists, beta antagonists, angiotensin-converting-enzyme inhibitor and Angiotensin II receptor blockers, Calcium ion antagonists, diuretics, and other drugs.

**Intraoperative blood pressure**

Recording frequency of intraoperative blood pressure values and heart rate value was one time/minute. Due to daily practical reasons, if the patient was sent out of the operating room, blood pressure will continue to be recorded. This blood pressure was extremely low or extremely high, or with a consecutive identical pattern. This data was not valid. Data washing was required. We used the following methods for data washing: first we removed non-physiological data, we defined (systolic blood pressure <20 mmHg and >300 mmHg and DBP <20 mmHg and >200 mmHg, HR<30 and >250 bpm) as non-physiological data, and deleted.^2^ To prevent invalid data from being mixed in, if the consecutive identical value exceeds 2 times in the data, the value will be removed afterwards till a different value.

**Anesthesiologist experience**

Working years of primary anesthesiologist were divided into ≤ 2, 2-5, 5-10,10-15,>15 years subgroups.

**Cancer or malignant tumor**

Using ICD-10 codes as below:

C00-C14 (Malignant neoplasms of lip, oral cavity and pharynx); C15-C26 Malignant neoplasms of digestive organs); C30-C39 (Malignant neoplasms of respiratory and intrathoracic organs); C40-C41 (Malignant neoplasms of bone and articular cartilage); C43-C44 (Melanoma and other malignant neoplasms of skin); C45-C49 (Malignant neoplasms of mesothelial and soft tissue); C50-C50 (Malignant neoplasms of breast); C51-C58 (Malignant neoplasms of female genital organs); C60-C63 (Malignant neoplasms of male genital organs); C64-C68 (Malignant neoplasms of urinary tract); C69-C72 (Malignant neoplasms of eye, brain and other parts of central nervous system); C73-C75 (Malignant neoplasms of thyroid and other endocrine glands); C76-C80 (Malignant neoplasms of ill-defined, other secondary and unspecified sites); C7A-C7A (Malignant neuroendocrine tumors); C81-C96 (Malignant neoplasms of lymphoid, hematopoietic and related tissue); D00-D09 (In situ neoplasms).

**Supplementary Tables**

Table-S1 Surgery type using International Classification of Diseases, 9th Revision, Clinical Modification (ICD-9-CM) codes. All operations other than the ICD codes listed were defined as "other." Cardiac and obstetric surgeries was excluded by in hospital department record in the database.

| ICD-9-v3 code | Procedure |
| --- | --- |
| 8.01‑16.99, 18.01‑20.99, 21-29.99 | OPERATIONS ON THE EYE, EAR, NOSE, MOUTH, AND PHARYNX |
| 85-86.99 | OPERATIONS ON THE INTEGUMENTARY SYSTEM |
| 55.01-71.9 | OPERATIONS ON THE URINARY SYSTEM AND GENITAL ORGANS |
| 76.01-84.99 | OPERATIONS ON THE MUSCULOSKELETAL SYSTEM |
| 1.01-5.9 | OPERATIONS ON THE NERVOUS SYSTEM |
| 35‑39.99 | OPERATIONS ON THE CARDIOVASCULAR SYSTEM |
| 42.01‑54.99 | OPERATIONS ON THE DIGESTIVE SYSTEM |
| 30.01-34.99 | OPERATIONS ON THE RESPIRATORY SYSTEM |

Figure-S1. Flowchart of the study

121474 surgeries among 109060 unique patients performed from July 1, 2012 to Dec 31, 2017

105009 surgeries excluded

8768 patients aged <18 y

12714 emergency surgeries

1710 cardiac surgeries

4871 obstetric surgeries

6801 kidney surgeries

3841 under local infiltration or MAC

1543 with first SBP < 90 mmHg

81226 eligible surgeries among 74519 unique patients reviewed

24087 surgeries excluded

3273 second surgeries within 1 year

17170 without serum cholesterol

60772 eligible surgeries among 57983 unique patients included

58123 surgeries without AKI

2649 surgeries with AKI

Table S2. Baseline Characteristics of the Patients Before and After propensity score weighting^a^

| Characteristic | Observed Data (N = 60772) | | | Propensity score Weighted Data (n = 116470.5) | | |
| --- | --- | --- | --- | --- | --- | --- |
|  | Lower HDL  (HDL >1.03)  (n =38622) | Higher HDL  (HDL <1.03)  (n = 22150) | Standardized  Difference b | Lower HDL  (HDL >1.03)  (n =61131.9) | Higher HDL  (HDL <1.03)  (n=55338.6) | Standardized  Difference |
| Male | 22824.0 (59.1) | 6793.0 (30.7) | 59.6 | 29612.4 (48.4) | 24826.1 (44.9) | 7.2 |
| Age, year |  |  | 13.4 |  |  | 2.2 |
| <50 | 14028.0 (36.3) | 6751.0 (30.5) |  | 21208.9 (34.7) | 18676.5 (33.7) |  |
| 50-60 | 8643.0 (22.4) | 5047.0 (22.8) |  | 13589.8 (22.2) | 12494.4 (22.6) |  |
| 60-70 | 8655.0 (22.4) | 5403.0 (24.4) |  | 14186.2 (23.2) | 12831.8 (23.2) |  |
| >70 | 7296.0 (18.9) | 4949.0 (22.3) |  | 12147.0 (19.9) | 11335.9 (20.5) |  |
| LDL (mmol/l) |  |  | 26.9 |  |  | 2.1 |
| < 2.20 | 8394.0 (21.7) | 6858.0 (31.0) |  | 15267.1 (25.0) | 13584.9 (24.5) |  |
| 2.20-2.71 | 9528.0 (24.7) | 5856.0 (26.4) |  | 15250.3 (24.9) | 13703.0 (24.8) |  |
| 2.71-3.24 | 9816.0 (25.4) | 5238.0 (23.6) |  | 15219.0 (24.9) | 13618.1 (24.6) |  |
| >3.24 | 10884.0 (28.2) | 4198.0 (19.0) |  | 15395.5 (25.2) | 14432.6 (26.1) |  |
| TCHO (mmol/l) |  |  | 50.7 |  |  | 4.9 |
| <3.93 | 7168.0 (18.6) | 8228.0 (37.1) |  | 15652.5 (25.6) | 15276.9 (27.6) |  |
| 3.93-4.54 | 9350.0 (24.2) | 5886.0 (26.6) |  | 15347.5 (25.1) | 13900.9 (25.1) |  |
| 4.54-5.19 | 10397.0 (26.9) | 4688.0 (21.2) |  | 15159.8 (24.8) | 13029.2 (23.5) |  |
| >5.19 | 11707.0 (30.3) | 3348.0 (15.1) |  | 14972.0 (24.5) | 13131.5 (23.7) |  |
| TG (mmol/l) |  |  | 67.8 | 15171.5 (24.8) | 11543.0 (20.9) | 9.7 |
| < 0.86 | 12518.0 (32.4) | 2687.0 (12.1) |  | 15178.1 (24.8) | 13914.1 (25.1) |  |
| 0.86-1.22 | 10741.0 (27.8) | 4500.0 (20.3) |  | 15239.0 (24.9) | 14698.2 (26.6) |  |
| 1.22-1.76 | 9051.0 (23.4) | 6133.0 (27.7) |  | 15543.3 (25.4) | 15183.3 (27.4) |  |
| >1.76 | 6312.0 (16.3) | 8830.0 (39.9) |  | 15171.5 (24.8) | 11543.0 (20.9) |  |
| Comorbidities |  |  |  |  |  |  |
| Hypertension | 9575.0 (24.8) | 7431.0 (33.5) | 19.4 | 16911.2 (27.7) | 16072.2 (29.0) | 3.1 |
| Coronary artery disease | 1571.0 (4.1) | 1614.0 (7.3) | 13.9 | 3218.9 (5.3) | 2951.0 (5.3) | 0.3 |
| Congestive heart failure | 259.0 (0.7) | 289.0 (1.3) | 6.4 | 557.4 (0.9) | 614.3 (1.1) | 2.0 |
| stroke | 1248.0 (3.2) | 1232.0 (5.6) | 11.4 | 2487.5 (4.1) | 2358.7 (4.3) | 1.0 |
| Diabetes mellitus | 3594.0 (9.3) | 3769.0 (17.0) | 23.0 | 7252.4 (11.9) | 7068.8 (12.8) | 2.8 |
| Renal insufficiency | 320.0 (0.8) | 487.0 (2.2) | 11.2 | 746.6 (1.2) | 766.0 (1.4) | 1.4 |
| Cancer surgery | 17847.0 (46.2) | 10684.0 (48.2) | 4.1 | 28454.6 (46.5) | 25658.9 (46.4) | 0.4 |
| Revised CRIc |  |  | 33.4 |  |  | 3.1 |
| 0 | 28577.0 (74.0) | 13225.0 (59.7) |  | 42195.2 (69.0) | 37495.7 (67.8) |  |
| 1 | 8167.0 (21.1) | 6386.0 (28.8) |  | 14599.8 (23.9) | 13570.3 (24.5) |  |
| 2 | 1524.0 (3.9) | 1875.0 (8.5) |  | 3383.9 (5.5) | 3312.7 (6.0) |  |
| ≥3 | 354.0 (0.9) | 664.0 (3.0) |  | 952.9 (1.6) | 959.9 (1.7) |  |
| Surgery |  |  | 16.8 |  |  | 9.2 |
| Eye/ear | 2073.0 (5.4) | 938.0 (4.2) |  | 3389.2 (5.5) | 2374.5 (4.3) |  |
| Integumentary | 1712.0 (4.4) | 491.0 (2.2) |  | 2202.5 (3.6) | 1795.2 (3.2) |  |
| Genital/urinary | 10113.0 (26.2) | 5719.0 (25.8) |  | 16025.9 (26.2) | 14150.3 (25.6) |  |
| Musculoskeletal | 4758.0 (12.3) | 2520.0 (11.4) |  | 7314.8 (12.0) | 6797.1 (12.3) |  |
| Nervous | 2049.0 (5.3) | 1442.0 (6.5) |  | 3571.2 (5.8) | 3195.9 (5.8) |  |
| Vascular | 1380.0 (3.6) | 831.0 (3.8) |  | 2204.0 (3.6) | 1887.3 (3.4) |  |
| Digestive | 12551.0 (32.5) | 7967.0 (36.0) |  | 19947.9 (32.6) | 19818.3 (35.8) |  |
| Respiratory | 2107.0 (5.5) | 1392.0 (6.3) |  | 3831.6 (6.3) | 2932.0 (5.3) |  |
| Other | 1879.0 (4.9) | 850.0 (3.8) |  | 2644.6 (4.3) | 2388.1 (4.3) |  |
| Complexity of surgery |  |  | 18.0 |  |  | 1.4 |
| low | 12667.0 (32.8) | 6779.0 (30.6) |  | 19589.8 (32.0) | 17383.0 (31.4) |  |
| Medium | 20892.0 (54.1) | 10998.0 (49.7) |  | 32084.0 (52.5) | 29224.8 (52.8) |  |
| High | 5063.0 (13.1) | 4373.0 (19.7) |  | 9458.1 (15.5) | 8730.9 (15.8) |  |
| primary anesthesiologist experience, year |  |  | 6.3 |  |  | 1.8 |
| >15 | 7530.0 (19.5) | 4084.0 (18.4) |  | 11739.8 (19.2) | 10715.5 (19.4) |  |
| 0-1 | 6429.0 (16.6) | 3651.0 (16.5) |  | 10161.7 (16.6) | 8890.7 (16.1) |  |
| 2-5 | 9331.0 (24.2) | 5536.0 (25.0) |  | 14949.3 (24.5) | 13447.0 (24.3) |  |
| 5-10 | 9533.0 (24.7) | 5139.0 (23.2) |  | 14779.4 (24.2) | 13485.6 (24.4) |  |
| 10-15 | 5799.0 (15.0) | 3740.0 (16.9) |  | 9501.7 (15.5) | 8799.8 (15.9) |  |
| Surgery duration min |  |  | 25 |  |  | 3.4 |
| <120 | 26118.0 (67.6) | 12720.0 (57.4) |  | 39137.7 (64.0) | 34531.3 (62.4) |  |
| 120-240 | 9006.0 (23.3) | 5853.0 (26.4) |  | 14979.6 (24.5) | 14074.6 (25.4) |  |
| 240-480 | 3187.0 (8.3) | 3151.0 (14.2) |  | 6273.3 (10.3) | 6041.0 (10.9) |  |
| >480 | 311.0 (0.8) | 426.0 (1.9) |  | 741.3 (1.2) | 691.7 (1.2) |  |

a: All values are reported as No. (%) unless otherwise specified.

b: Standardized differences compare imbalance among variables without being affected by sample size. Standardized differences of less than 10% are Considered by some authors to indicate good balance between groups.

c: cardiac risk index.

LDL: low density lipoprotein cholesterol

TCHO: total cholesterol

TG: triglyceride

Table S3. Multivariate logistic regression detecting association of HDL and postoperative AKI after propensity score weighting

| Factors | Odds ratio | P value |
| --- | --- | --- |
| HDL, 0.96 - 1.14 (mmol/l) | reference |  |
| < 0.96 | 1.28 (1.14 - 1.41) | <.001 |
| 1.14 - 1.35 | 0.91 (0.80 - 1.03) | 0.15 |
| > 1.35 | 0.75 (0.64 - 0.85) | <.001 |
| statin | 0.79 (0.53 - 1.05) | 0.168 |
| Female to male | 0.66 (0.58 - 0.73) | <.001 |
| Revised cardiac index 0 | reference |  |
| 1 | 1.55 (1.37 - 1.72) | <.001 |
| 2 | 3.53 (3.01 - 4.04) | <.001 |
| >=3 | 7.54 (6.11 - 8.97) | <.001 |
| Anesthesia type (general) | reference |  |
| General + epidural | 1.19 (0.98 - 1.40) | 0.049 |
| General + nerve block | 0.63 (0.43 - 0.84) | 0.005 |
| Intrathecal | 0.72 (0.61 - 0.83) | <.001 |
| nerve block | 0.89 (0.56 - 1.23) | 0.555 |
| Surgical duration (< 120 minutes) | reference |  |
| 120 - 240 | 1.17 (1.04 - 1.30) | 0.007 |
| 240 - 480 | 1.21 (1.03 - 1.40) | 0.013 |
| > 480 | 1.61 (1.14 - 2.08) | 0.001 |
| Cancer to benign surgery | 1.95 (1.76 - 2.14) | <.001 |
| Hypertension | 1.17 (1.05 - 1.28) | 0.002 |
| Anemia | 1.95 (1.65 - 2.26) | <.001 |
| Intraoperative hypotension | 1.54 (0.96 - 2.12) | 0.023 |
| Body mass index, (18.5 - 23 kg/m^2^) | reference |  |
| < 18.5 | 1.48 (1.20 - 1.76) | <.001 |
| 23.0 - 27.5 | 0.89 (0.80 - 0.97) | 0.016 |
| > 27.5 | 0.97 (0.85 - 1.09) | 0.634 |
| Intraoperative blood transfusion | 1.22 (1.06 - 1.38) | 0.002 |
| complexity of surgery (low) | reference |  |
| medium | 1.96 (1.74 - 2.18) | <.001 |
| high | 0.64 (0.53 - 0.75) | <.001 |
| anesthesiologist experience, > 15 year | reference |  |
| 0 - 1 | 1.07 (0.92 - 1.22) | 0.346 |
| 2 - 5 | 1.13 (0.98 - 1.27) | 0.068 |
| 5 - 10 | 0.89 (0.77 - 1.01) | 0.08 |
| 10 -15 | 1.26 (1.09 - 1.44) | 0.001 |
| Preoperative albumin > 33.4 | 0.55 (0.50 - 0.61) | <.001 |
| Age (<40 year) | reference |  |
| 40 - 50 | 1.29 (1.06 - 1.53) | 0.005 |
| 50 - 60 | 1.65 (1.38 - 1.92) | <.001 |
| 60 - 70 | 1.51 (1.25 - 1.76) | <.001 |
| > 70 | 1.58 (1.30 - 1.85) | <.001 |
| Dexmedetomidine use | 0.83 (0.76 - 0.91) | <.001 |
| Colloid use | 1.11 (0.99 - 1.23) | 0.049 |
| Preoperative creatine, < 69.4 umol/L | reference |  |
| 69.4 - 80.2 | 0.59 (0.51 - 0.68) | <.001 |
| 80.2 - 92.5 | 0.68 (0.58 - 0.78) | <.001 |
| > 92.5 | 1.39 (1.20 - 1.58) | <.001 |

Table S4. Outcome of the Patients Before and After propensity score weighting^a^

| Characteristic | Observed Data (N = 60772) | | | Propensity score Weighted Data (n = 116470.5) | | |
| --- | --- | --- | --- | --- | --- | --- |
|  | Lower HDL  (HDL >1.03)  (n =38622) | Higher HDL  (HDL <1.03)  (n = 22150) | P value | Lower HDL  (HDL >1.03)  (n =61131.9) | Higher HDL  (HDL <1.03)  (n=55338.6) | P value |
| All | 1144.0 (3.0) | 1505.0 (6.8) | <0.001 | 2323.2 (3.8) | 2834.0 (5.1) | <0.001 |
| III | 101.0 (0.5) | 204.0 (1.3) | <0.001 | 217.6 (0.6) | 330.3 (0.9) | 0.003 |
| II | 66.0 (0.3) | 86.0 (0.6) | <0.001 | 139.9 (0.4) | 178.5 (0.5) | 0.255 |
| I | 977.0 (4.4) | 1215.0 (8.0) | <0.001 | 1965.6 (5.3) | 2325.2 (6.5) | 0.001 |

a: All values are reported as No. (%) unless otherwise specified.

Table S5. Sensitivity analysis with different cut values of HDL levels for main outcome.

| HDL Cut values (mmol/l) | Odds ratio before propensity score weighting | P value | Odds ratio after propensity score weighting | P value |
| --- | --- | --- | --- | --- |
| HDL <1.03 vs ≥ 1.03 | 1.40 (1.27~1.52) | <.001 | 1.32 (1.21 - 1.46) | <.001 |
| HDL <0.96 vs ≥ 0.96 | 1.39 (1.27~1.52) | <.001 | 1.29 (1.17~1.43) | <.001 |
| HDL <1.14 vs ≥ 1.14 | 1.36 (1.23~1.49) | <.001 | 1.25 (1.13~1.40) | <.001 |
| HDL <1.35 vs ≥ 1.35 | 1.30 (1.15 - 1.48) | <.001 | 1.32 (1.15~1.52) | <.001 |

Table-S5. Complexity of surgery using Modified John Hopkins hospital criteria (MJHSC)

Here listed grade 3 and grade 1 code, MJHSC grade 2 ICD-9-v3 procedure code was defined by excluding grade1 and grade 3.

MJHSC grade3 ICD-9 procedure code

| ICD9 code | Description |
| --- | --- |
| 00.70 | Revision of hip replacement, both acetabular and femoral components |
| 00.71 | Revision of hip replacement, acetabular component |
| 00.72 | Revision of hip replacement, femoral component |
| 00.74 | Hip bearing surface, metal-on-polyethylene |
| 00.75 | Hip bearing surface, metal-on-metal |
| 00.76 | Hip bearing surface, ceramic-on-ceramic |
| 00.77 | Hip bearing surface, ceramic-on-polyethylene |
| 00.80 | Revision of knee replacement, total (all components) |
| 00.81 | Revision of knee replacement, tibial component |
| 00.82 | Revision of knee replacement, femoral component |
| 00.83 | Revision of knee replacement, patellar component |
| 00.84 | Revision of total knee replacement, tibial insert (liner) |
| 00.85 | Resurfacing hip, total, acetabulum and femoral head |
| 00.86 | Resurfacing hip, partial, femoral head |
| 00.87 | Resurfacing hip, partial, acetabulum |
| 00.91 | Transplant from live related donor |
| 00.92 | Transplant from live non-related donor |
| 00.93 | Transplant from cadaver |
| 01.22 | Removal of intracranial neurostimulator lead(s) |
| 01.24 | Other craniotomy |
| 01.31 | Incision of cerebral meninges |
| 01.32 | Lobotomy and tractotomy |
| 01.39 | Other incision of brain |
| 01.41 | Operations on thalamus |
| 01.42 | Operations on globus pallidus |
| 01.51 | Excision of lesion or tissue of cerebral meninges |
| 01.52 | Hemispherectomy |
| 01.53 | Lobectomy of brain |
| 02.11 | Simple suture of dura mater of brain |
| 02.14 | Choroid plexectomy |
| 02.91 | Lysis of cortical adhesions |
| 02.92 | Repair of brain |
| 02.99 | Other operations on skull, brain, and cerebral meninges |
| 03.09 | Other exploration and decompression of spinal canal |
| 03.51 | Repair of spinal meningocele |
| 03.52 | Repair of spinal myelomeningocele |
| 03.53 | Repair of vertebral fracture |
| 03.59 | Other repair and plastic operations on spinal cord structures |
| 04.41 | Decompression of trigeminal nerve root |
| 04.42 | Other cranial nerve decompression |
| 04.5 | Cranial or peripheral nerve graft |
| 04.75 | Revision of previous repair of cranial and peripheral nerves |
| 07.51 | Exploration of pineal field |
| 07.52 | Incision of pineal gland |
| 07.53 | Partial excision of pineal gland |
| 07.54 | Total excision of pineal gland |
| 07.59 | Other operations on pineal gland |
| 07.61 | Partial excision of pituitary gland, transfrontal approach |
| 07.62 | Partial excision of pituitary gland, transsphenoidal approach |
| 07.63 | Partial excision of pituitary gland, unspecified approach |
| 07.64 | Total excision of pituitary gland, transfrontal approach |
| 07.65 | Total excision of pituitary gland, transsphenoidal approach |
| 07.68 | Total excision of pituitary gland, other specified approach |
| 07.69 | Total excision of pituitary gland, unspecified approach |
| 07.71 | Exploration of pituitary fossa |
| 07.72 | Incision of pituitary gland |
| 07.79 | Other operations on hypophysis |
| 07.82 | Other total excision of thymus |
| 27.69 | Other plastic repair of palate |
| 29.4 | Plastic operation on pharynx |
| 29.53 | Closure of other fistula of pharynx |
| 30.29 | Other partial laryngectomy |
| 30.3 | Complete laryngectomy |
| 30.4 | Radical laryngectomy |
| 31.69 | Other repair of larynx |
| 31.79 | Other repair and plastic operations on trachea |
| 31.98 | Other operations on larynx |
| 32.09 | Other local excision or destruction of lesion or tissue of bronchus |
| 32.20 | Thoracoscopic excision of lesion or tissue of lung |
| 32.23 | Open ablation of lung lesion or tissue |
| 32.25 | Thoracoscopic ablation of lung lesion or tissue |
| 32.28 | Endoscopic excision or destruction of lesion or tissue of lung |
| 32.29 | Other local excision or destruction of lesion or tissue of lung |
| 32.30 | Thoracoscopic segmental resection of lung |
| 32.39 | Other and unspecified segmental resection of lung |
| 32.41 | Thoracoscopic lobectomy of lung |
| 32.49 | Other lobectomy of lung |
| 32.50 | Thoracoscopic pneumonectomy |
| 32.59 | Other and unspecified pneumonectomy |
| 32.6 | Radical dissection of thoracic structures |
| 33.1 | Incision of lung |
| 33.39 | Other surgical collapse of lung |
| 33.41 | Suture of laceration of bronchus |
| 33.42 | Closure of bronchial fistula |
| 33.43 | Closure of laceration of lung |
| 33.48 | Other repair and plastic operations on bronchus |
| 33.49 | Other repair and plastic operations on lung |
| 33.50 | Lung transplantation, not otherwise specified |
| 33.51 | Unilateral lung transplantation |
| 33.52 | Bilateral lung transplantation |
| 33.6 | Combined heart-lung transplantation |
| 34.02 | Exploratory thoracotomy |
| 34.03 | Reopening of recent thoracotomy site |
| 34.05 | Creation of pleuroperitoneal shunt |
| 34.1 | Incision of mediastinum |
| 34.3 | Excision or destruction of lesion or tissue of mediastinum |
| 34.51 | Decortication of lung |
| 34.59 | Other excision of pleura |
| 34.6 | Scarification of pleura |
| 34.73 | Closure of other fistula of thorax |
| 34.92 | Injection into thoracic cavity |
| 34.93 | Repair of pleura |
| 34.99 | Other operations on thorax |
| 35.03 | Closed heart valvotomy, pulmonary valve |
| 35.08 | Transapical replacement of pulmonary valve |
| 35.10 | Open heart valvuloplasty without replacement, unspecified valve |
| 35.11 | Open heart valvuloplasty of aortic valve without replacement |
| 35.12 | Open heart valvuloplasty of mitral valve without replacement |
| 35.13 | Open heart valvuloplasty of pulmonary valve without replacement |
| 35.14 | Open heart valvuloplasty of tricuspid valve without replacement |
| 35.20 | Open and other replacement of unspecified heart valve |
| 35.21 | Open and other replacement of aortic valve with tissue graft |
| 35.22 | Open and other replacement of aortic valve |
| 35.23 | Open and other replacement of mitral valve with tissue graft |
| 35.24 | Open and other replacement of mitral valve |
| 35.25 | Open and other replacement of pulmonary valve with tissue graft |
| 35.26 | Open and other replacement of pulmonary valve |
| 35.27 | Open and other replacement of tricuspid valve with tissue graft |
| 35.28 | Open and other replacement of tricuspid valve |
| 35.31 | Operations on papillary muscle |
| 35.32 | Operations on chordae tendineae |
| 35.33 | Annuloplasty |
| 35.34 | Infundibulectomy |
| 35.35 | Operations on trabeculae carneae cordis |
| 35.39 | Operations on other structures adjacent to valves of heart |
| 35.41 | Enlargement of existing atrial septal defect |
| 35.50 | Repair of unspecified septal defect of heart with prosthesis |
| 35.51 | Repair of atrial septal defect with prosthesis, open technique |
| 35.52 | Repair of atrial septal defect with prosthesis, closed technique |
| 35.53 | Repair of ventricular septal defect with prosthesis, open technique |
| 35.54 | Repair of endocardial cushion defect with prosthesis |
| 35.55 | Repair of ventricular septal defect with prosthesis, closed technique |
| 35.60 | Repair of unspecified septal defect of heart with tissue graft |
| 35.61 | Repair of atrial septal defect with tissue graft |
| 35.62 | Repair of ventricular septal defect with tissue graft |
| 35.63 | Repair of endocardial cushion defect with tissue graft |
| 35.70 | Other and unspecified repair of unspecified septal defect of heart |
| 35.71 | Other and unspecified repair of atrial septal defect |
| 35.72 | Other and unspecified repair of ventricular septal defect |
| 35.73 | Other and unspecified repair of endocardial cushion defect |
| 35.81 | Total repair of tetralogy of fallot |
| 35.82 | Total repair of total anomalous pulmonary venous connection |
| 35.83 | Total repair of truncus arteriosus |
| 35.84 | Total correction of transposition of great vessels, not elsewhere classified |
| 35.91 | Interatrial transposition of venous return |
| 35.92 | Creation of conduit between right ventricle and pulmonary artery |
| 35.93 | Creation of conduit between left ventricle and aorta |
| 35.94 | Creation of conduit between atrium and pulmonary artery |
| 35.95 | Revision of corrective procedure on heart |
| 35.96 | Percutaneous balloon valvuloplasty |
| 35.97 | Percutaneous mitral valve repair with implant |
| 35.98 | Other operations on septa of heart |
| 35.99 | Other operations on valves of heart |
| 36.03 | Open chest coronary artery angioplasty |
| 36.10 | Aortocoronary bypass for heart revascularization, not otherwise specified |
| 36.11 | (Aorto)coronary bypass of one coronary artery |
| 36.12 | (Aorto)coronary bypass of two coronary arteries |
| 36.13 | (Aorto)coronary bypass of three coronary arteries |
| 36.14 | (Aorto)coronary bypass of four or more coronary arteries |
| 36.15 | Single internal mammary-coronary artery bypass |
| 36.16 | Double internal mammary-coronary artery bypass |
| 36.17 | Abdominal-coronary artery bypass |
| 36.19 | Other bypass anastomosis for heart revascularization |
| 36.2 | Heart revascularization by arterial implant |
| 36.31 | Open chest transmyocardial revascularization |
| 36.32 | Other transmyocardial revascularization |
| 36.33 | Endoscopic transmyocardial revascularization |
| 36.34 | Percutaneous transmyocardial revascularization |
| 36.39 | Other heart revascularization |
| 36.91 | Repair of aneurysm of coronary vessel |
| 36.99 | Other operations on vessels of heart |
| 37.0 | Pericardiocentesis |
| 37.10 | Incision of heart, not otherwise specified |
| 37.11 | Cardiotomy |
| 37.12 | Pericardiotomy |
| 37.32 | Excision of aneurysm of heart |
| 37.35 | Partial ventriculectomy |
| 37.36 | Excision, destruction, or exclusion of left atrial appendage (LAA) |
| 37.37 | Excision or destruction of other lesion or tissue of heart, thoracoscopic approach |
| 37.49 | Other repair of heart and pericardium |
| 37.51 | Heart transplantation |
| 37.52 | Implantation of total internal biventricular heart replacement system |
| 37.53 | Replacement or repair of thoracic unit of (total) replacement heart system |
| 37.54 | Replacement or repair of other implantable component of (total) replacement heart system |
| 37.55 | Removal of internal biventricular heart replacement system |
| 37.60 | Implantation or insertion of biventricular external heart assist system |
| 37.91 | Open chest cardiac massage |
| 38.02 | Incision of vessel, other vessels of head and neck |
| 38.03 | Incision of vessel, upper limb vessels |
| 38.04 | Incision of vessel, aorta |
| 38.05 | Incision of vessel, other thoracic vessels |
| 38.06 | Incision of vessel, abdominal arteries |
| 38.07 | Incision of vessel, abdominal veins |
| 38.08 | Incision of vessel, lower limb arteries |
| 38.09 | Incision of vessel, lower limb veins |
| 38.10 | Endarterectomy, unspecified site |
| 38.11 | Endarterectomy, intracranial vessels |
| 38.12 | Endarterectomy, other vessels of head and neck |
| 38.13 | Endarterectomy, upper limb vessels |
| 38.14 | Endarterectomy, aorta |
| 38.15 | Endarterectomy, other thoracic vessels |
| 38.16 | Endarterectomy, abdominal arteries |
| 38.18 | Endarterectomy, lower limb arteries |
| 38.30 | Resection of vessel with anastomosis, unspecified site |
| 38.31 | Resection of vessel with anastomosis, intracranial vessels |
| 38.32 | Resection of vessel with anastomosis, other vessels of head and neck |
| 38.33 | Resection of vessel with anastomosis, upper limb vessels |
| 38.34 | Resection of vessel with anastomosis, aorta |
| 38.35 | Resection of vessel with anastomosis, other thoracic vessels |
| 38.36 | Resection of vessel with anastomosis, abdominal arteries |
| 38.37 | Resection of vessel with anastomosis, abdominal veins |
| 38.38 | Resection of vessel with anastomosis, lower limb arteries |
| 38.39 | Resection of vessel with anastomosis, lower limb veins |
| 38.40 | Resection of vessel with replacement, unspecified site |
| 38.41 | Resection of vessel with replacement, intracranial vessels |
| 38.42 | Resection of vessel with replacement, other vessels of head and neck |
| 38.43 | Resection of vessel with replacement, upper limb vessels |
| 38.44 | Resection of vessel with replacement, aorta, abdominal |
| 38.45 | Resection of vessel with replacement, thoracic vessels |
| 38.46 | Resection of vessel with replacement, abdominal arteries |
| 38.47 | Resection of vessel with replacement, abdominal veins |
| 38.48 | Resection of vessel with replacement, lower limb arteries |
| 38.49 | Resection of vessel with replacement, lower limb veins |
| 38.61 | Other excision of vessels, intracranial vessels |
| 38.62 | Other excision of vessels, other vessels of head and neck |
| 38.63 | Other excision of vessels, upper limb vessels |
| 38.64 | Other excision of vessels, aorta, abdominal |
| 38.65 | Other excision of vessels, thoracic vessels |
| 38.66 | Other excision of vessels, abdominal arteries |
| 38.67 | Other excision of vessels, abdominal veins |
| 38.68 | Other excision of vessels, lower limb arteries |
| 38.7 | Interruption of the vena cava |
| 38.81 | Other surgical occlusion of vessels, intracranial vessels |
| 39.0 | Systemic to pulmonary artery shunt |
| 39.21 | Caval-pulmonary artery anastomosis |
| 39.22 | Aorta-subclavian-carotid bypass |
| 39.23 | Other intrathoracic vascular shunt or bypass |
| 39.24 | Aorta-renal bypass |
| 39.25 | Aorta-iliac-femoral bypass |
| 39.26 | Other intra-abdominal vascular shunt or bypass |
| 39.27 | Arteriovenostomy for renal dialysis |
| 39.28 | Extracranial-intracranial (EC-IC) vascular bypass |
| 39.31 | Suture of artery |
| 39.51 | Clipping of aneurysm |
| 39.52 | Other repair of aneurysm |
| 39.54 | Re-entry operation (aorta) |
| 39.56 | Repair of blood vessel with tissue patch graft |
| 39.57 | Repair of blood vessel with synthetic patch graft |
| 39.58 | Repair of blood vessel with unspecified type of patch graft |
| 39.59 | Other repair of vessel |
| 39.74 | Endovascular removal of obstruction from head and neck vessel(s) |
| 39.79 | Other endovascular procedures on other vessels |
| 39.84 | Revision of carotid sinus stimulation lead(s) only |
| 39.85 | Revision of carotid sinus stimulation pulse generator |
| 39.86 | Removal of carotid sinus stimulation device, total system |
| 39.87 | Removal of carotid sinus stimulation lead(s) only |
| 39.88 | Removal of carotid sinus stimulation pulse generator only |
| 39.89 | Other operations on carotid body, carotid sinus and other vascular bodies |
| 40.59 | Radical excision of other lymph nodes |
| 40.69 | Other operations on thoracic duct |
| 42.09 | Other incision of esophagus |
| 42.10 | Esophagostomy, not otherwise specified |
| 42.12 | Exteriorization of esophageal pouch |
| 42.21 | Operative esophagoscopy by incision |
| 42.31 | Local excision of esophageal diverticulum |
| 42.32 | Local excision of other lesion or tissue of esophagus |
| 42.41 | Partial esophagectomy |
| 42.42 | Total esophagectomy |
| 42.51 | Intrathoracic esophagoesophagostomy |
| 42.52 | Intrathoracic esophagogastrostomy |
| 42.53 | Intrathoracic esophageal anastomosis with interposition of small bowel |
| 42.55 | Intrathoracic esophageal anastomosis with interposition of colon |
| 42.58 | Intrathoracic esophageal anastomosis with other interposition |
| 42.59 | Other intrathoracic anastomosis of esophagus |
| 42.61 | Antesternal esophagoesophagostomy |
| 42.62 | Antesternal esophagogastrostomy |
| 42.63 | Antesternal esophageal anastomosis with interposition of small bowel |
| 42.64 | Other antesternal esophagoenterostomy |
| 42.65 | Antesternal esophageal anastomosis with interposition of colon |
| 42.7 | Esophagomyotomy |
| 42.82 | Suture of laceration of esophagus |
| 42.84 | Repair of esophageal fistula, not elsewhere classified |
| 42.85 | Repair of esophageal stricture |
| 42.91 | Ligation of esophageal varices |
| 43.5 | Partial gastrectomy with anastomosis to esophagus |
| 43.6 | Partial gastrectomy with anastomosis to duodenum |
| 43.7 | Partial gastrectomy with anastomosis to jejunum |
| 43.91 | Total gastrectomy with intestinal interposition |
| 43.99 | Other total gastrectomy |
| 44.29 | Other pyloroplasty |
| 44.38 | Laparoscopic gastroenterostomy |
| 44.39 | Other gastroenterostomy without gastrectomy |
| 44.5 | Revision of gastric anastomosis |
| 44.65 | Esophagogastroplasty |
| 44.66 | Other procedures for creation of esophagogastric sphincteric competence |
| 44.91 | Ligation of gastric varices |
| 45.81 | Laparoscopic total intra-abdominal colectomy |
| 45.82 | Open total intra-abdominal colectomy |
| 45.91 | Small-to-small intestinal anastomosis |
| 45.92 | Anastomosis of small intestine to rectal stump |
| 45.93 | Other small-to-large intestinal anastomosis |
| 45.94 | Large-to-large intestinal anastomosis |
| 45.95 | Anastomosis to anus |
| 46.79 | Other repair of intestine |
| 46.93 | Revision of anastomosis of small intestine |
| 46.94 | Revision of anastomosis of large intestine |
| 48.51 | Laparoscopic abdominoperineal resection of the rectum |
| 48.52 | Open abdominoperineal resection of the rectum |
| 48.74 | Rectorectostomy |
| 51.31 | Anastomosis of gallbladder to hepatic ducts |
| 51.32 | Anastomosis of gallbladder to intestine |
| 51.34 | Anastomosis of gallbladder to stomach |
| 51.36 | Choledochoenterostomy |
| 51.37 | Anastomosis of hepatic duct to gastrointestinal tract |
| 51.39 | Other bile duct anastomosis |
| 51.49 | Incision of other bile ducts for relief of obstruction |
| 51.72 | Choledochoplasty |
| 51.79 | Repair of other bile ducts |
| 51.83 | Pancreatic sphincteroplasty |
| 51.94 | Revision of anastomosis of biliary tract |
| 52.01 | Drainage of pancreatic cyst by catheter |
| 52.09 | Other pancreatotomy |
| 52.13 | Endoscopic retrograde pancreatography [ERP] |
| 52.21 | Endoscopic excision or destruction of lesion or tissue of pancreatic duct |
| 52.22 | Other excision or destruction of lesion or tissue of pancreas or pancreatic duct |
| 52.3 | Marsupialization of pancreatic cyst |
| 52.51 | Proximal pancreatectomy |
| 52.52 | Distal pancreatectomy |
| 52.53 | Radical subtotal pancreatectomy |
| 52.59 | Other partial pancreatectomy |
| 52.6 | Total pancreatectomy |
| 52.7 | Radical pancreaticoduodenectomy |
| 52.81 | Reimplantation of pancreatic tissue |
| 52.82 | Homotransplant of pancreas |
| 52.83 | Heterotransplant of pancreas |
| 52.84 | Autotransplantation of cells of Islets of Langerhans |
| 52.85 | Allotransplantation of cells of Islets of Langerhans |
| 52.92 | Cannulation of pancreatic duct |
| 52.95 | Other repair of pancreas |
| 52.96 | Anastomosis of pancreas |
| 54.94 | Creation of peritoneovascular shunt |
| 54.95 | Incision of peritoneum |
| 55.86 | Anastomosis of kidney |
| 55.87 | Correction of ureteropelvic junction |
| 56.71 | Urinary diversion to intestine |
| 56.72 | Revision of ureterointestinal anastomosis |
| 56.73 | Nephrocystanastomosis, not otherwise specified |
| 56.74 | Ureteroneocystostomy |
| 56.75 | Transureteroureterostomy |
| 56.89 | Other repair of ureter |
| 57.79 | Other total cystectomy |
| 57.85 | Cystourethroplasty and plastic repair of bladder neck |
| 57.86 | Repair of bladder exstrophy |
| 57.87 | Reconstruction of urinary bladder |
| 57.88 | Other anastomosis of bladder |
| 63.82 | Reconstruction of surgically divided vas deferens |
| 63.83 | Epididymovasostomy |
| 64.44 | Reconstruction of penis |
| 65.73 | Other salpingo-oophoroplasty |
| 65.76 | Laparoscopic salpingo-oophoroplasty |
| 65.79 | Other repair of ovary |
| 66.72 | Salpingo-oophorostomy |
| 66.73 | Salpingo-salpingostomy |
| 66.74 | Salpingo-uterostomy |
| 66.79 | Other repair of fallopian tube |
| 67.69 | Other repair of cervix |
| 69.23 | Vaginal repair of chronic inversion of uterus |
| 70.61 | Vaginal construction |
| 70.62 | Vaginal reconstruction |
| 71.79 | Other repair of vulva and perineum |
| 76.41 | Total mandibulectomy with synchronous reconstruction |
| 76.44 | Total ostectomy of other facial bone with synchronous reconstruction |
| 77.29 | Wedge osteotomy, other bones |
| 77.99 | Total ostectomy, other bones |
| 81.01 | Atlas-axis spinal fusion |
| 81.02 | Other cervical fusion of the anterior column, anterior technique |
| 81.03 | Other cervical fusion of the posterior column, posterior technique |
| 81.04 | Dorsal and dorsolumbar fusion of the anterior column, anterior technique |
| 81.05 | Dorsal and dorsolumbar fusion of the posterior column, posterior technique |
| 81.06 | Lumbar and lumbosacral fusion of the anterior column, anterior technique |
| 81.08 | Lumbar and lumbosacral fusion of the anterior column, posterior technique |
| 81.31 | Refusion of atlas-axis spine |
| 81.32 | Refusion of other cervical spine, anterior column, anterior technique |
| 81.33 | Refusion of other cervical spine, posterior column, posterior technique |
| 81.34 | Refusion of dorsal and dorsolumbar spine, anterior column, anterior technique |
| 81.35 | Refusion of dorsal and dorsolumbar spine, posterior column, posterior technique |
| 81.36 | Refusion of lumbar and lumbosacral spine, anterior column, anterior technique |
| 81.37 | Refusion of lumbar and lumbosacral spine, posterior column, posterior technique |
| 81.38 | Refusion of lumbar and lumbosacral spine, anterior column, posterior technique |
| 81.51 | Total hip replacement |
| 81.52 | Partial hip replacement |
| 81.53 | Revision of hip replacement, not otherwise specified |
| 81.54 | Total knee replacement |
| 81.55 | Revision of knee replacement, not otherwise specified |
| 81.62 | Fusion or refusion of 2-3 vertebrae |
| 81.63 | Fusion or refusion of 4-8 vertebrae |
| 81.64 | Fusion or refusion of 9 or more vertebrae |
| 81.80 | Other total shoulder replacement |
| 81.81 | Partial shoulder replacement |
| 84.51 | Insertion of interbody spinal fusion device |
| 84.59 | Insertion of other spinal devices |
| 84.61 | Insertion of partial spinal disc prosthesis, cervical |
| 84.62 | Insertion of total spinal disc prosthesis, cervical |
| 84.63 | Insertion of spinal disc prosthesis, thoracic |
| 84.64 | Insertion of partial spinal disc prosthesis, lumbosacral |
| 84.65 | Insertion of total spinal disc prosthesis, lumbosacral |
| 84.66 | Revision or replacement of artificial spinal disc prosthesis, cervical |
| 84.67 | Revision or replacement of artificial spinal disc prosthesis, thoracic |
| 84.68 | Revision or replacement of artificial spinal disc prosthesis, lumbosacral |
| 85.70 | Total reconstruction of breast, not otherwise specified |
| 85.79 | Other total reconstruction of breast |

MJHSC grade 1 ICD-9 procedure code

| 01.11 | Closed [percutaneous] [needle] biopsy of cerebral meninges |
| --- | --- |
| 01.12 | Open biopsy of cerebral meninges |
| 01.13 | Closed [percutaneous] [needle] biopsy of brain |
| 01.14 | Open biopsy of brain |
| 01.15 | Biopsy of skull |
| 01.25 | Other craniectomy |
| 01.26 | Insertion of catheter(s) into cranial cavity or tissue |
| 01.27 | Removal of catheter(s) from cranial cavity or tissue |
| 01.29 | Removal of cranial neurostimulator pulse generator |
| 01.59 | Other excision or destruction of lesion or tissue of brain |
| 02.02 | Elevation of skull fracture fragments |
| 03.32 | Biopsy of spinal cord or spinal meninges |
| 04.11 | Closed [percutaneous] [needle] biopsy of cranial or peripheral nerve or ganglion |
| 04.12 | Open biopsy of cranial or peripheral nerve or ganglion |
| 04.43 | Release of carpal tunnel |
| 05.11 | Biopsy of sympathetic nerve or ganglion |
| 06.11 | Closed [percutaneous] [needle] biopsy of thyroid gland |
| 06.12 | Open biopsy of thyroid gland |
| 06.13 | Biopsy of parathyroid gland |
| 07.11 | Closed [percutaneous] [needle] biopsy of adrenal gland |
| 08.01 | Incision of lid margin |
| 08.02 | Severing of blepharorrhaphy |
| 08.09 | Other incision of eyelid |
| 08.11 | Biopsy of eyelid |
| 08.19 | Other diagnostic procedures on eyelid |
| 08.20 | Removal of lesion of eyelid, not otherwise specified |
| 08.21 | Excision of chalazion |
| 08.22 | Excision of other minor lesion of eyelid |
| 08.23 | Excision of major lesion of eyelid, partial-thickness |
| 08.24 | Excision of major lesion of eyelid, full-thickness |
| 08.25 | Destruction of lesion of eyelid |
| 08.31 | Repair of blepharoptosis by frontalis muscle technique with suture |
| 08.32 | Repair of blepharoptosis by frontalis muscle technique with fascial sling |
| 08.33 | Repair of blepharoptosis by resection or advancement of levator muscle or aponeurosis |
| 08.34 | Repair of blepharoptosis by other levator muscle techniques |
| 08.35 | Repair of blepharoptosis by tarsal technique |
| 08.36 | Repair of blepharoptosis by other techniques |
| 08.38 | Correction of lid retraction |
| 08.41 | Repair of entropion or ectropion by thermocauterization |
| 08.42 | Repair of entropion or ectropion by suture technique |
| 08.43 | Repair of entropion or ectropion with wedge resection |
| 08.49 | Other repair of entropion or ectropion |
| 08.51 | Canthotomy |
| 08.52 | Blepharorrhaphy |
| 08.59 | Other adjustment of lid position |
| 08.81 | Linear repair of laceration of eyelid or eyebrow |
| 08.89 | Other eyelid repair |
| 08.93 | Other epilation of eyelid |
| 09.0 | Incision of lacrimal gland |
| 09.11 | Biopsy of lacrimal gland |
| 09.12 | Biopsy of lacrimal sac |
| 09.19 | Other diagnostic procedures on lacrimal system |
| 09.20 | Excision of lacrimal gland, not otherwise specified |
| 09.41 | Probing of lacrimal punctum |
| 09.42 | Probing of lacrimal canaliculi |
| 09.43 | Probing of nasolacrimal duct |
| 09.44 | Intubation of nasolacrimal duct |
| 09.49 | Other manipulation of lacrimal passage |
| 09.51 | Incision of lacrimal punctum |
| 09.52 | Incision of lacrimal canaliculi |
| 09.53 | Incision of lacrimal sac |
| 09.59 | Other incision of lacrimal passages |
| 09.6 | Excision of lacrimal sac and passage |
| 09.71 | Correction of everted punctum |
| 09.72 | Other repair of punctum |
| 09.73 | Repair of canaliculus |
| 09.81 | Dacryocystorhinostomy [DCR] |
| 09.91 | Obliteration of lacrimal punctum |
| 09.99 | Other operations on lacrimal system |
| 10.0 | Removal of embedded foreign body from conjunctiva by incision |
| 10.1 | Other incision of conjunctiva |
| 10.21 | Biopsy of conjunctiva |
| 10.29 | Other diagnostic procedures on conjunctiva |
| 10.31 | Excision of lesion or tissue of conjunctiva |
| 10.32 | Destruction of lesion of conjunctiva |
| 10.33 | Other destructive procedures on conjunctiva |
| 10.42 | Reconstruction of conjunctival cul-de-sac with free graft |
| 10.5 | Lysis of adhesions of conjunctiva and eyelid |
| 10.6 | Repair of laceration of conjunctiva |
| 10.91 | Subconjunctival injection |
| 10.99 | Other operations on conjunctiva |
| 11.0 | Magnetic removal of embedded foreign body from cornea |
| 11.1 | Incision of cornea |
| 11.21 | Scraping of cornea for smear or culture |
| 11.22 | Biopsy of cornea |
| 11.29 | Other diagnostic procedures on cornea |
| 11.39 | Other excision of pterygium |
| 11.41 | Mechanical removal of corneal epithelium |
| 11.42 | Thermocauterization of corneal lesion |
| 11.43 | Cryotherapy of corneal lesion |
| 11.49 | Other removal or destruction of corneal lesion |
| 11.51 | Suture of corneal laceration |
| 11.91 | Tattooing of cornea |
| 11.99 | Other operations on cornea |
| 12.01 | Removal of intraocular foreign body from anterior segment of eye with use of magnet |
| 12.11 | Iridotomy with transfixion |
| 12.12 | Other iridotomy |
| 12.21 | Diagnostic aspiration of anterior chamber of eye |
| 12.22 | Biopsy of iris |
| 12.51 | Goniopuncture without goniotomy |
| 12.52 | Goniotomy without goniopuncture |
| 15.01 | Biopsy of extraocular muscle or tendon |
| 16.23 | Biopsy of eyeball and orbit |
| 16.89 | Other repair of injury of eyeball or orbit |
| 17.11 | Laparoscopic repair of direct inguinal hernia with graft or prosthesis |
| 17.12 | Laparoscopic repair of indirect inguinal hernia with graft or prosthesis |
| 17.21 | Laparoscopic bilateral repair of direct inguinal hernia with graft or prosthesis |
| 17.22 | Laparoscopic bilateral repair of indirect inguinal hernia with graft or prosthesis |
| 17.23 | Laparoscopic bilateral repair of inguinal hernia, one direct and one indirect, with graft or prosthesis |
| 18.12 | Biopsy of external ear |
| 18.29 | Excision or destruction of other lesion of external ear |
| 18.6 | Reconstruction of external auditory canal |
| 18.71 | Construction of auricle of ear |
| 18.79 | Other plastic repair of external ear |
| 18.9 | Other operations on external ear |
| 19.3 | Other operations on ossicular chain |
| 19.4 | Myringoplasty |
| 20.01 | Myringotomy with insertion of tube |
| 20.09 | Other myringotomy |
| 20.1 | Removal of tympanostomy tube |
| 20.32 | Biopsy of middle and inner ear |
| 20.41 | Simple mastoidectomy |
| 20.59 | Other excision of middle ear |
| 20.92 | Revision of mastoidectomy |
| 21.22 | Biopsy of nose |
| 21.32 | Local excision or destruction of other lesion of nose |
| 21.83 | Total nasal reconstruction |
| 21.86 | Limited rhinoplasty |
| 21.99 | Other operations on nose |
| 22.11 | Closed [endoscopic] [needle] biopsy of nasal sinus |
| 22.9 | Other operations on nasal sinuses |
| 24.11 | Biopsy of gum |
| 25.02 | Open biopsy of tongue |
| 26.11 | Closed [needle] biopsy of salivary gland or duct |
| 26.12 | Open biopsy of salivary gland or duct |
| 26.49 | Other repair and plastic operations on salivary gland or duct |
| 27.21 | Biopsy of bony palate |
| 27.22 | Biopsy of uvula and soft palate |
| 27.23 | Biopsy of lip |
| 27.24 | Biopsy of mouth, unspecified structure |
| 27.43 | Other excision of lesion or tissue of lip |
| 27.57 | Attachment of pedicle or flap graft to lip and mouth |
| 27.59 | Other plastic repair of mouth |
| 28.0 | Incision and drainage of tonsil and peritonsillar structures |
| 28.11 | Biopsy of tonsils and adenoids |
| 28.2 | Tonsillectomy without adenoidectomy |
| 28.3 | Tonsillectomy with adenoidectomy |
| 28.5 | Excision of lingual tonsil |
| 28.7 | Control of hemorrhage after tonsillectomy and adenoidectomy |
| 28.92 | Excision of lesion of tonsil and adenoid |
| 29.12 | Pharyngeal biopsy |
| 31.1 | Temporary tracheostomy |
| 31.21 | Mediastinal tracheostomy |
| 31.29 | Other permanent tracheostomy |
| 31.3 | Other incision of larynx or trachea |
| 31.41 | Tracheoscopy through artificial stoma |
| 31.43 | Closed [endoscopic] biopsy of larynx |
| 31.44 | Closed [endoscopic] biopsy of trachea |
| 31.45 | Open biopsy of larynx or trachea |
| 31.63 | Revision of laryngostomy |
| 31.72 | Closure of external fistula of trachea |
| 31.74 | Revision of tracheostomy |
| 31.75 | Reconstruction of trachea and construction of artificial larynx |
| 31.95 | Tracheoesophageal fistulization |
| 32.01 | Endoscopic excision or destruction of lesion or tissue of bronchus |
| 33.22 | Fiber-optic bronchoscopy |
| 33.23 | Other bronchoscopy |
| 33.24 | Closed [endoscopic] biopsy of bronchus |
| 33.25 | Open biopsy of bronchus |
| 33.26 | Closed [percutaneous] [needle] biopsy of lung |
| 33.27 | Closed endoscopic biopsy of lung |
| 33.71 | Endoscopic insertion or replacement of bronchial valve(s), single lobe |
| 33.73 | Endoscopic insertion or replacement of bronchial valve(s), multiple lobes |
| 33.78 | Endoscopic removal of bronchial device(s) or substances |
| 33.79 | Endoscopic insertion of other bronchial device or substances |
| 34.01 | Incision of chest wall |
| 34.04 | Insertion of intercostal catheter for drainage |
| 34.20 | Thoracoscopic pleural biopsy |
| 34.23 | Biopsy of chest wall |
| 34.24 | Other pleural biopsy |
| 34.25 | Closed [percutaneous] [needle] biopsy of mediastinum |
| 34.26 | Open mediastinal biopsy |
| 34.27 | Biopsy of diaphragm |
| 34.4 | Excision or destruction of lesion of chest wall |
| 34.71 | Suture of laceration of chest wall |
| 34.72 | Closure of thoracostomy |
| 34.79 | Other repair of chest wall |
| 37.25 | Biopsy of heart |
| 37.79 | Revision or relocation of cardiac device pocket |
| 38.21 | Biopsy of blood vessel |
| 38.50 | Ligation and stripping of varicose veins, unspecified site |
| 38.51 | Ligation and stripping of varicose veins, intracranial vessels |
| 38.52 | Ligation and stripping of varicose veins, other vessels of head and neck |
| 38.53 | Ligation and stripping of varicose veins, upper limb vessels |
| 38.55 | Ligation and stripping of varicose veins, other thoracic vessels |
| 38.59 | Ligation and stripping of varicose veins, lower limb veins |
| 38.60 | Other excision of vessels, unspecified site |
| 38.69 | Other excision of vessels, lower limb veins |
| 38.80 | Other surgical occlusion of vessels, unspecified site |
| 38.93 | Venous catheterization, not elsewhere classified |
| 39.1 | Intra-abdominal venous shunt |
| 39.29 | Other (peripheral) vascular shunt or bypass |
| 40.11 | Biopsy of lymphatic structure |
| 40.23 | Excision of axillary lymph node |
| 40.24 | Excision of inguinal lymph node |
| 40.54 | Radical groin dissection |
| 41.31 | Biopsy of bone marrow |
| 41.32 | Closed [aspiration] [percutaneous] biopsy of spleen |
| 41.33 | Open biopsy of spleen |
| 42.11 | Cervical esophagostomy |
| 42.19 | Other external fistulization of esophagus |
| 42.24 | Closed [endoscopic] biopsy of esophagus |
| 42.25 | Open biopsy of esophagus |
| 42.83 | Closure of esophagostomy |
| 43.19 | Other gastrostomy |
| 44.12 | Gastroscopy through artificial stoma |
| 44.14 | Closed [endoscopic] biopsy of stomach |
| 44.15 | Open biopsy of stomach |
| 44.62 | Closure of gastrostomy |
| 45.12 | Endoscopy of small intestine through artificial stoma |
| 45.14 | Closed [endoscopic] biopsy of small intestine |
| 45.15 | Open biopsy of small intestine |
| 45.16 | Esophagogastroduodenoscopy [EGD] with closed biopsy |
| 45.22 | Endoscopy of large intestine through artificial stoma |
| 45.25 | Closed [endoscopic] biopsy of large intestine |
| 45.26 | Open biopsy of large intestine |
| 46.10 | Colostomy, not otherwise specified |
| 46.11 | Temporary colostomy |
| 46.13 | Permanent colostomy |
| 46.14 | Delayed opening of colostomy |
| 46.21 | Temporary ileostomy |
| 46.23 | Other permanent ileostomy |
| 46.24 | Delayed opening of ileostomy |
| 46.39 | Other enterostomy |
| 46.41 | Revision of stoma of small intestine |
| 46.42 | Repair of pericolostomy hernia |
| 46.43 | Other revision of stoma of large intestine |
| 46.51 | Closure of stoma of small intestine |
| 46.52 | Closure of stoma of large intestine |
| 47.91 | Appendicostomy |
| 48.1 | Proctostomy |
| 48.24 | Closed [endoscopic] biopsy of rectum |
| 48.25 | Open biopsy of rectum |
| 48.26 | Biopsy of perirectal tissue |
| 48.62 | Anterior resection of rectum with synchronous colostomy |
| 48.72 | Closure of proctostomy |
| 49.22 | Biopsy of perianal tissue |
| 49.23 | Biopsy of anus |
| 49.39 | Other local excision or destruction of lesion or tissue of anus |
| 49.46 | Excision of hemorrhoids |
| 49.49 | Other procedures on hemorrhoids |
| 49.99 | Other operations on anus |
| 50.11 | Closed (percutaneous) [needle] biopsy of liver |
| 50.12 | Open biopsy of liver |
| 50.14 | Laparoscopic liver biopsy |
| 50.19 | Other diagnostic procedures on liver |
| 51.12 | Percutaneous biopsy of gallbladder or bile ducts |
| 51.13 | Open biopsy of gallbladder or bile ducts |
| 51.14 | Other closed [endoscopic] biopsy of biliary duct or sphincter of Oddi |
| 51.85 | Endoscopic sphincterotomy and papillotomy |
| 51.88 | Endoscopic removal of stone(s) from biliary tract |
| 51.92 | Closure of cholecystostomy |
| 52.11 | Closed [aspiration] [needle] [percutaneous] biopsy of pancreas |
| 52.12 | Open biopsy of pancreas |
| 52.14 | Closed [endoscopic] biopsy of pancreatic duct |
| 52.4 | Internal drainage of pancreatic cyst |
| 52.94 | Endoscopic removal of stone(s) from pancreatic duct |
| 53.00 | Unilateral repair of inguinal hernia, not otherwise specified |
| 53.01 | Other and open repair of direct inguinal hernia |
| 53.02 | Other and open repair of indirect inguinal hernia |
| 53.03 | Other and open repair of direct inguinal hernia with graft or prosthesis |
| 53.04 | Other and open repair of indirect inguinal hernia with graft or prosthesis |
| 53.05 | Repair of inguinal hernia with graft or prosthesis, not otherwise specified |
| 53.10 | Bilateral repair of inguinal hernia, not otherwise specified |
| 53.11 | Other and open bilateral repair of direct inguinal hernia |
| 53.12 | Other and open bilateral repair of indirect inguinal hernia |
| 53.13 | Other and open bilateral repair of inguinal hernia, one direct and one indirect |
| 53.14 | Other and open bilateral repair of direct inguinal hernia with graft or prosthesis |
| 53.15 | Other and open bilateral repair of indirect inguinal hernia with graft or prosthesis |
| 53.16 | Other and open bilateral repair of inguinal hernia, one direct and one indirect, with graft or prosthesis |
| 53.17 | Bilateral inguinal hernia repair with graft or prosthesis, not otherwise specified |
| 53.21 | Unilateral repair of femoral hernia with graft or prosthesis |
| 53.29 | Other unilateral femoral herniorrhaphy |
| 53.31 | Bilateral repair of femoral hernia with graft or prosthesis |
| 53.39 | Other bilateral femoral herniorrhaphy |
| 53.41 | Other and open repair of umbilical hernia with graft or prosthesis |
| 53.42 | Laparoscopic repair of umbilical hernia with graft or prosthesis |
| 53.43 | Other laparoscopic umbilical herniorrhaphy |
| 53.49 | Other open umbilical herniorrhaphy |
| 53.51 | Incisional hernia repair |
| 53.59 | Repair of other hernia of anterior abdominal wall |
| 53.61 | Other open incisional hernia repair with graft or prosthesis |
| 53.69 | Other and open repair of other hernia of anterior abdominal wall with graft or prosthesis |
| 53.71 | Laparoscopic repair of diaphragmatic hernia, abdominal approach |
| 53.72 | Other and open repair of diaphragmatic hernia, abdominal approach |
| 53.80 | Repair of diaphragmatic hernia with thoracic approach, not otherwise specified |
| 53.83 | Laparoscopic repair of diaphragmatic hernia, with thoracic approach |
| 53.9 | Other hernia repair |
| 54.0 | Incision of abdominal wall |
| 54.22 | Biopsy of abdominal wall or umbilicus |
| 54.23 | Biopsy of peritoneum |
| 54.24 | Closed [percutaneous] [needle] biopsy of intra-abdominal mass |
| 54.3 | Excision or destruction of lesion or tissue of abdominal wall or umbilicus |
| 54.93 | Creation of cutaneoperitoneal fistula |
| 55.02 | Nephrostomy |
| 55.03 | Percutaneous nephrostomy without fragmentation |
| 55.04 | Percutaneous nephrostomy with fragmentation |
| 55.11 | Pyelotomy |
| 55.12 | Pyelostomy |
| 55.21 | Nephroscopy |
| 55.23 | Closed [percutaneous] [needle] biopsy of kidney |
| 55.24 | Open biopsy of kidney |
| 55.39 | Other local destruction or excision of renal lesion or tissue |
| 55.82 | Closure of nephrostomy and pyelostomy |
| 55.92 | Percutaneous aspiration of kidney (pelvis) |
| 56.0 | Transurethral removal of obstruction from ureter and renal pelvis |
| 56.2 | Ureterotomy |
| 56.31 | Ureteroscopy |
| 56.32 | Closed percutaneous biopsy of ureter |
| 56.33 | Closed endoscopic biopsy of ureter |
| 56.34 | Open biopsy of ureter |
| 56.41 | Partial ureterectomy |
| 56.51 | Formation of cutaneous uretero-ileostomy |
| 56.52 | Revision of cutaneous uretero-ileostomy |
| 56.61 | Formation of other cutaneous ureterostomy |
| 56.83 | Closure of ureterostomy |
| 56.91 | Dilation of ureteral meatus |
| 57.0 | Transurethral clearance of bladder |
| 57.17 | Percutaneous cystostomy |
| 57.18 | Other suprapubic cystostomy |
| 57.21 | Vesicostomy |
| 57.31 | Cystoscopy through artificial stoma |
| 57.32 | Other cystoscopy |
| 57.33 | Closed [transurethral] biopsy of bladder |
| 57.34 | Open biopsy of bladder |
| 57.41 | Transurethral lysis of intraluminal adhesions |
| 57.49 | Other transurethral excision or destruction of lesion or tissue of bladder |
| 57.82 | Closure of cystostomy |
| 57.91 | Sphincterotomy of bladder |
| 57.92 | Dilation of bladder neck |
| 58.0 | Urethrotomy |
| 58.1 | Urethral meatotomy |
| 58.21 | Perineal urethroscopy |
| 58.22 | Other urethroscopy |
| 58.23 | Biopsy of urethra |
| 58.24 | Biopsy of periurethral tissue |
| 58.31 | Endoscopic excision or destruction of lesion or tissue of urethra |
| 58.39 | Other local excision or destruction of lesion or tissue of urethra |
| 58.41 | Suture of laceration of urethra |
| 58.42 | Closure of urethrostomy |
| 58.44 | Reanastomosis of urethra |
| 58.45 | Repair of hypospadias or epispadias |
| 58.46 | Other reconstruction of urethra |
| 58.5 | Release of urethral stricture |
| 58.6 | Dilation of urethra |
| 58.91 | Incision of periurethral tissue |
| 58.92 | Excision of periurethral tissue |
| 58.93 | Implantation of artificial urinary sphincter [AUS] |
| 58.99 | Other operations on urethra and periurethral tissue |
| 59.21 | Biopsy of perirenal or perivesical tissue |
| 59.4 | Suprapubic sling operation |
| 59.5 | Retropubic urethral suspension |
| 59.6 | Paraurethral suspension |
| 59.71 | Levator muscle operation for urethrovesical suspension |
| 59.8 | Ureteral catheterization |
| 59.93 | Replacement of ureterostomy tube |
| 59.94 | Replacement of cystostomy tube |
| 59.95 | Ultrasonic fragmentation of urinary stones |
| 60.0 | Incision of prostate |
| 60.11 | Closed [percutaneous] [needle] biopsy of prostate |
| 60.12 | Open biopsy of prostate |
| 60.13 | Closed [percutaneous] biopsy of seminal vesicles |
| 60.14 | Open biopsy of seminal vesicles |
| 60.15 | Biopsy of periprostatic tissue |
| 60.19 | Other diagnostic procedures on seminal vesicles |
| 60.21 | Transurethral (ultrasound) guided laser induced prostatectomy (TULIP) |
| 60.29 | Other transurethral prostatectomy |
| 60.72 | Incision of seminal vesicle |
| 60.95 | Transurethral balloon dilation of the prostatic urethra |
| 60.97 | Other transurethral destruction of prostate tissue by other thermotherapy |
| 61.11 | Biopsy of scrotum or tunica vaginalis |
| 61.3 | Excision or destruction of lesion or tissue of scrotum |
| 62.11 | Closed [percutaneous] [needle] biopsy of testis |
| 62.12 | Open biopsy of testis |
| 63.01 | Biopsy of spermatic cord, epididymis, or vas deferens |
| 63.6 | Vasotomy |
| 63.99 | Other operations on spermatic card, epididymis, and vas deferens |
| 64.0 | Circumcision |
| 64.11 | Biopsy of penis |
| 64.2 | Local excision or destruction of lesion of penis |
| 64.91 | Dorsal or lateral slit of prepuce |
| 65.01 | Laparoscopic oophorotomy |
| 65.09 | Other oophorotomy |
| 65.11 | Aspiration biopsy of ovary |
| 65.12 | Other biopsy of ovary |
| 65.13 | Laparoscopic biopsy of ovary |
| 66.02 | Salpingostomy |
| 66.11 | Biopsy of fallopian tube |
| 66.29 | Other bilateral endoscopic destruction or occlusion of fallopian tubes |
| 66.8 | Insufflation of fallopian tube |
| 67.11 | Endocervical biopsy |
| 67.12 | Other cervical biopsy |
| 67.2 | Conization of cervix |
| 67.32 | Destruction of lesion of cervix by cauterization |
| 67.33 | Destruction of lesion of cervix by cryosurgery |
| 67.39 | Other excision or destruction of lesion or tissue of cervix |
| 67.4 | Amputation of cervix |
| 67.51 | Transabdominal cerclage of cervix |
| 67.59 | Other repair of internal cervical os |
| 67.61 | Suture of laceration of cervix |
| 67.62 | Repair of fistula of cervix |
| 68.12 | Hysteroscopy |
| 68.13 | Open biopsy of uterus |
| 68.14 | Open biopsy of uterine ligaments |
| 68.15 | Closed biopsy of uterine ligaments |
| 68.16 | Closed biopsy of uterus |
| 68.21 | Division of endometrial synechiae |
| 68.22 | Incision or excision of congenital septum of uterus |
| 68.23 | Endometrial ablation |
| 68.29 | Other excision or destruction of lesion of uterus |
| 69.02 | Dilation and curettage following delivery or abortion |
| 69.09 | Other dilation and curettage |
| 69.49 | Other repair of uterus |
| 69.51 | Aspiration curettage of uterus for termination of pregnancy |
| 69.52 | Aspiration curettage following delivery or abortion |
| 69.59 | Other aspiration curettage of uterus |
| 69.91 | Insertion of therapeutic device into uterus |
| 69.95 | Incision of cervix |
| 69.96 | Removal of cerclage material from cervix |
| 70.14 | Other vaginotomy |
| 70.23 | Biopsy of cul-de-sac |
| 70.24 | Vaginal biopsy |
| 71.11 | Biopsy of vulva |
| 71.29 | Other operations on Bartholin's gland |
| 71.3 | Other local excision or destruction of vulva and perineum |
| 73.93 | Incision of cervix to assist delivery |
| 74.3 | Removal of extratubal ectopic pregnancy |
| 75.33 | Fetal blood sampling and biopsy |
| 76.11 | Biopsy of facial bone |
| 77.04 | Sequestrectomy, carpals and metacarpals |
| 77.12 | Other incision of bone without division, humerus |
| 77.24 | Wedge osteotomy, carpals and metacarpals |
| 77.41 | Biopsy of bone, scapula, clavicle, and thorax [ribs and sternum] |
| 77.42 | Biopsy of bone, humerus |
| 77.43 | Biopsy of bone, radius and ulna |
| 77.44 | Biopsy of bone, carpals and metacarpals |
| 77.45 | Biopsy of bone, femur |
| 77.46 | Biopsy of bone, patella |
| 77.47 | Biopsy of bone, tibia and fibula |
| 77.48 | Biopsy of bone, tarsals and metatarsals |
| 77.49 | Biopsy of bone, other bones |
| 77.64 | Local excision of lesion or tissue of bone, carpals and metacarpals |
| 77.84 | Other partial ostectomy, carpals and metacarpals |
| 78.04 | Bone graft, carpals and metacarpals |
| 78.14 | Application of external fixator device, carpals and metacarpals |
| 78.34 | Limb lengthening procedures, carpals and metacarpals |
| 78.44 | Other repair or plastic operations on bone, carpals and metacarpals |
| 78.46 | Other repair or plastic operations on bone, patella |
| 78.54 | Internal fixation of bone without fracture reduction, carpals and metacarpals |
| 78.57 | Internal fixation of bone without fracture reduction, tibia and fibula |
| 78.60 | Removal of implanted devices from bone, unspecified site |
| 78.61 | Removal of implanted devices from bone, scapula, clavicle, and thorax [ribs and sternum] |
| 78.62 | Removal of implanted devices from bone, humerus |
| 78.63 | Removal of implanted devices from bone, radius and ulna |
| 78.64 | Removal of implanted devices from bone, carpals and metacarpals |
| 78.65 | Removal of implanted devices from bone, femur |
| 78.66 | Removal of implanted devices from bone, patella |
| 78.67 | Removal of implanted devices from bone, tibia and fibula |
| 78.68 | Removal of implanted devices from bone, tarsals and metatarsals |
| 78.70 | Osteoclasis, unspecified site |
| 78.71 | Osteoclasis, scapula, clavicle, and thorax [ribs and sternum] |
| 78.72 | Osteoclasis, humerus |
| 78.73 | Osteoclasis, radius and ulna |
| 78.74 | Osteoclasis, carpals and metacarpals |
| 78.75 | Osteoclasis, femur |
| 78.76 | Osteoclasis, patella |
| 78.77 | Osteoclasis, tibia and fibula |
| 78.78 | Osteoclasis, tarsals and metatarsals |
| 79.03 | Closed reduction of fracture without internal fixation, carpals and metacarpals |
| 79.13 | Closed reduction of fracture with internal fixation, carpals and metacarpals |
| 79.23 | Open reduction of fracture without internal fixation, carpals and metacarpals |
| 79.33 | Open reduction of fracture with internal fixation, carpals and metacarpals |
| 79.61 | Debridement of open fracture site, humerus |
| 79.62 | Debridement of open fracture site, radius and ulna |
| 79.63 | Debridement of open fracture site, carpals and metacarpals |
| 79.64 | Debridement of open fracture site, phalanges of hand |
| 79.65 | Debridement of open fracture site, femur |
| 79.66 | Debridement of open fracture site, tibia and fibula |
| 79.67 | Debridement of open fracture site, tarsals and metatarsals |
| 79.68 | Debridement of open fracture site, phalanges of foot |
| 79.69 | Debridement of open fracture site, other specified bone |
| 80.11 | Other arthrotomy, shoulder |
| 80.12 | Other arthrotomy, elbow |
| 80.13 | Other arthrotomy, wrist |
| 80.15 | Other arthrotomy, hip |
| 80.16 | Other arthrotomy, knee |
| 80.17 | Other arthrotomy, ankle |
| 80.18 | Other arthrotomy, foot and toe |
| 80.21 | Arthroscopy, shoulder |
| 80.22 | Arthroscopy, elbow |
| 80.23 | Arthroscopy, wrist |
| 80.24 | Arthroscopy, hand and finger |
| 80.25 | Arthroscopy, hip |
| 80.26 | Arthroscopy, knee |
| 80.27 | Arthroscopy, ankle |
| 80.28 | Arthroscopy, foot and toe |
| 80.31 | Biopsy of joint structure, shoulder |
| 80.32 | Biopsy of joint structure, elbow |
| 80.33 | Biopsy of joint structure, wrist |
| 80.34 | Biopsy of joint structure, hand and finger |
| 80.35 | Biopsy of joint structure, hip |
| 80.36 | Biopsy of joint structure, knee |
| 80.37 | Biopsy of joint structure, ankle |
| 80.39 | Biopsy of joint structure, other specified sites |
| 80.41 | Division of joint capsule, ligament, or cartilage, shoulder |
| 80.42 | Division of joint capsule, ligament, or cartilage, elbow |
| 80.43 | Division of joint capsule, ligament, or cartilage, wrist |
| 80.46 | Division of joint capsule, ligament, or cartilage, knee |
| 80.51 | Excision of intervertebral disc |
| 80.52 | Intervertebral chemonucleolysis |
| 80.59 | Other destruction of intervertebral disc |
| 80.6 | Excision of semilunar cartilage of knee |
| 80.71 | Synovectomy, shoulder |
| 80.72 | Synovectomy, elbow |
| 80.73 | Synovectomy, wrist |
| 80.74 | Synovectomy, hand and finger |
| 80.75 | Synovectomy, hip |
| 80.76 | Synovectomy, knee |
| 80.77 | Synovectomy, ankle |
| 80.78 | Synovectomy, foot and toe |
| 80.81 | Other local excision or destruction of lesion of joint, shoulder |
| 80.82 | Other local excision or destruction of lesion of joint, elbow |
| 80.83 | Other local excision or destruction of lesion of joint, wrist |
| 80.84 | Other local excision or destruction of lesion of joint, hand and finger |
| 80.85 | Other local excision or destruction of lesion of joint, hip |
| 80.86 | Other local excision or destruction of lesion of joint, knee |
| 80.87 | Other local excision or destruction of lesion of joint, ankle |
| 80.88 | Other local excision or destruction of lesion of joint, foot and toe |
| 81.11 | Ankle fusion |
| 81.13 | Subtalar fusion |
| 81.23 | Arthrodesis of shoulder |
| 81.40 | Repair of hip, not elsewhere classified |
| 81.45 | Other repair of the cruciate ligaments |
| 81.47 | Other repair of knee |
| 81.49 | Other repair of ankle |
| 81.93 | Suture of capsule or ligament of upper extremity |
| 81.94 | Suture of capsule or ligament of ankle and foot |
| 82.02 | Myotomy of hand |
| 82.03 | Bursotomy of hand |
| 82.31 | Bursectomy of hand |
| 82.89 | Other plastic operations on hand |
| 82.92 | Aspiration of bursa of hand |
| 82.93 | Aspiration of other soft tissue of hand |
| 82.94 | Injection of therapeutic substance into bursa of hand |
| 82.95 | Injection of therapeutic substance into tendon of hand |
| 83.02 | Myotomy |
| 83.03 | Bursotomy |
| 83.09 | Other incision of soft tissue |
| 83.11 | Achillotenotomy |
| 83.12 | Adductor tenotomy of hip |
| 83.19 | Other division of soft tissue |
| 83.21 | Open biopsy of soft tissue |
| 83.31 | Excision of lesion of tendon sheath |
| 83.39 | Excision of lesion of other soft tissue |
| 83.45 | Other myectomy |
| 83.88 | Other plastic operations on tendon |
| 83.91 | Lysis of adhesions of muscle, tendon, fascia, and bursa |
| 83.94 | Aspiration of bursa |
| 83.95 | Aspiration of other soft tissue |
| 83.96 | Injection of therapeutic substance into bursa |
| 83.97 | Injection of therapeutic substance into tendon |
| 83.98 | Injection of locally acting therapeutic substance into other soft tissue |
| 83.99 | Other operations on muscle, tendon, fascia, and bursa |
| 84.00 | Upper limb amputation, not otherwise specified |
| 84.01 | Amputation and disarticulation of finger |
| 84.02 | Amputation and disarticulation of thumb |
| 84.10 | Lower limb amputation, not otherwise specified |
| 84.11 | Amputation of toe |
| 85.11 | Closed [percutaneous] [needle] biopsy of breast |
| 85.12 | Open biopsy of breast |
| 85.20 | Excision or destruction of breast tissue, not otherwise specified |
| 85.43 | Unilateral extended simple mastectomy |
| 85.45 | Unilateral radical mastectomy |
| 85.71 | Latissimus dorsi myocutaneous flap |
| 85.72 | Transverse rectus abdominis myocutaneous (TRAM) flap, pedicled |
| 85.73 | Transverse rectus abdominis myocutaneous (TRAM) flap, free |
| 85.74 | Deep inferior epigastric artery perforator (DIEP) flap, free |
| 85.75 | Superficial inferior epigastric artery (SIEA) flap, free |
| 85.76 | Gluteal artery perforator (GAP) flap, free |
| 85.81 | Suture of laceration of breast |
| 85.84 | Pedicle graft to breast |
| 86.01 | Aspiration of skin and subcutaneous tissue |
| 86.02 | Injection or tattooing of skin lesion or defect |
| 86.04 | Other incision with drainage of skin and subcutaneous tissue |
| 86.05 | Incision with removal of foreign body or device from skin and subcutaneous tissue |
| 86.09 | Other incision of skin and subcutaneous tissue |
| 86.11 | Closed biopsy of skin and subcutaneous tissue |
| 86.22 | Excisional debridement of wound, infection, or burn |
| 86.24 | Chemosurgery of skin |
| 86.25 | Dermabrasion |
| 86.26 | Ligation of dermal appendage |
| 86.27 | Debridement of nail, nail bed, or nail fold |
| 86.28 | Nonexcisional debridement of wound, infection or burn |
| 86.3 | Other local excision or destruction of lesion or tissue of skin and subcutaneous tissue |
| 86.4 | Radical excision of skin lesion |
| 86.59 | Closure of skin and subcutaneous tissue of other sites |
| 86.62 | Other skin graft to hand |
| 86.65 | Heterograft to skin |
| 86.66 | Homograft to skin |
| 86.70 | Pedicle or flap graft, not otherwise specified |
| 86.71 | Cutting and preparation of pedicle grafts or flaps |
| 86.72 | Advancement of pedicle graft |
| 86.73 | Attachment of pedicle or flap graft to hand |
| 86.74 | Attachment of pedicle or flap graft to other sites |
| 86.75 | Revision of pedicle or flap graft |
| 86.82 | Facial rhytidectomy |
| 86.84 | Relaxation of scar or web contracture of skin |
| 86.89 | Other repair and reconstruction of skin and subcutaneous tissue |
| 86.91 | Excision of skin for graft |
| 86.92 | Electrolysis and other epilation of skin |
| 86.93 | Insertion of tissue expander |
| 87.38 | Sinogram of chest wall |
| 87.76 | Retrograde cystourethrogram |
| 88.38 | Other computerized axial tomography |
| 89.23 | Urethral sphincter electromyogram |
| 89.25 | Urethral pressure profile [UPP] |
| 96.24 | Dilation and manipulation of enterostomy stoma |
| 96.27 | Manual reduction of hernia |
| 96.28 | Manual reduction of enterostomy prolapse |
| 96.36 | Irrigation of gastrostomy or enterostomy |
| 96.41 | Irrigation of cholecystostomy and other biliary tube |
| 96.45 | Irrigation of nephrostomy and pyelostomy |
| 96.46 | Irrigation of ureterostomy and ureteral catheter |
| 96.47 | Irrigation of cystostomy |
| 96.55 | Tracheostomy toilette |
| 97.02 | Replacement of gastrostomy tube |
| 97.03 | Replacement of tube or enterostomy device of small intestine |
| 97.04 | Replacement of tube or enterostomy device of large intestine |
| 97.43 | Removal of sutures from thorax |
| 97.51 | Removal of gastrostomy tube |
| 97.54 | Removal of cholecystostomy tube |
| 97.61 | Removal of pyelostomy and nephrostomy tube |
| 97.62 | Removal of ureterostomy tube and ureteral catheter |
| 97.63 | Removal of cystostomy tube |
| 97.65 | Removal of urethral stent |
| 97.71 | Removal of intrauterine contraceptive device |
| 98.16 | Removal of intraluminal foreign body from uterus without incision |
| 98.18 | Removal of intraluminal foreign body from artificial stoma without incision |
| 98.19 | Removal of intraluminal foreign body from urethra without incision |
| 98.51 | Extracorporeal shockwave lithotripsy [ESWL] of the kidney, ureter and/or bladder |
| 98.52 | Extracorporeal shockwave lithotripsy [ESWL] of the gallbladder and/or bile duct |
| 98.59 | Extracorporeal shockwave lithotripsy of other sites |

**Reference**

1. Schwarze ML, Barnato AE, Rathouz PJ, et al. Development of a list of high-risk operations for patients 65 years and older. JAMA surgery 2015;150:325-31.

2. Monk TG, Bronsert MR, Henderson WG, et al. Association between intraoperative hypotension and hypertension and 30-day postoperative mortality in noncardiac surgery. The Journal of the American Society of Anesthesiologists 2015;123:307-19.
